# Chronic hypoxia for the adaptation of extracellular vesicle phenotype

**DOI:** 10.1038/s41598-024-73453-1

**Published:** 2024-10-24

**Authors:** Chris S. Pridgeon, Kerttu Airavaara, Julia Monola, Alisa Jokela, Daniel Palmer, Marjo Yliperttula, Riina Harjumäki

**Affiliations:** 1 Division of Pharmaceutical Biosciences, Drug Research Program, Faculty of Pharmacy, University of Helsinki, Helsinki, Finland; 2 Institut für Biostatistik und Informatik in Medizin und Alternsforschung, University of Rostock, Rostock, Germany

**Keywords:** Cell biology, Membrane trafficking

## Abstract

Variations in oxygen level affect the phenotype of cells and extracellular vesicles (EVs). Depending on the metabolic oxygen demand of cells, hypoxic cell culture can produce conditions more like those found in vivo, and with appropriate oxygen levels, mimic hypoxic tumours. However, most previous experiments studying both EVs and the effects of hypoxia on cells use periods of 72 h or less of hypoxia. We hypothesised that this was insufficient time for adaptation to hypoxic conditions both for EVs and cells which may skew the results of such studies. In this study, the effects of acute (72 h) and chronic hypoxia (> 2 weeks) on the phenotype of HepG2 and PC3 cells and their EVs were examined. Cells could be cultured normally under chronic hypoxic conditions and cryopreserved and recovered. The effects of hypoxia on EV phenotype are slow to establish and dependent on cell line. In PC3 cells, the greatest change in phenotype and increase in EV production occurred only with chronic hypoxic culture. In HepG2 cells, the number of EVs produced was insensitive to hypoxic culture and the greatest changes in protein expression were observed after acute hypoxic culture. Nonetheless, biphasic changes in EV phenotype were detected in both cell types in response to either acute or chronic hypoxia. These results indicate that for cells which do not induce consumptive oxygen depletion, prolonged hypoxic culture is required for complete adaptation.

**Supplementary Information:**

The online version contains supplementary material available at 10.1038/s41598-024-73453-1.

## Introduction

Accurately reproducing the in vivo cellular environment in vitro has been hypothesised, and in some cases shown, to increase the similarity of cultured cells to their in vivo counterparts. This can be observed in the trend in recent years to culture cells in three dimensions e.g. spheroids and organoids, with flow^1^ or in oxygen conditions more closely reproducing those found in vivo. Practically, this often means culturing cells in hypoxic conditions.

Hypoxia as it relates to cell culture is often loosely defined, for example, hypoxic cell culture is typically considered as culture in oxygen levels lower than those found in a standard cell incubator, i.e. air mixed with 5% CO_2_ in a humidified, 37 °C environment. This definition is imperfect, as even normoxic cultures may expose cells to conditions that are either physiologically hyperoxic or hypoxic depending on cell type and culture conditions such as medium depth^2^. For cell experiments, oxygen levels can be viewed from three perspectives: (1) *experimental*, where the oxygen level is judged relative to those found in a typical cell culture incubator, independent of the oxygen level cells are exposed to in vivo; (2) *physiological*, where the oxygen level is judged relative the levels for that cell type in vivo, sometimes referred to as physioxia; or (3) *cellular*, where the oxygen level is judged relative to a cell’s metabolic requirements, which may differ from physiological oxygen for example in some tumours. Thus, we can refer to, for example, experimental hyperoxia or physiological hypoxia. In this study, unless otherwise noted, “hypoxia” refers to experimental hypoxia.

The amount of oxygen cells experience in vitro is dependent on several factors: the concentration and pressure of oxygen in the environment, the cell’s metabolic oxygen demands and the depth of medium over the cells. For example, the metabolic activity of hepatocytes and hepatic cell lines produces near-anoxic conditions even in standard cell incubators, which is termed consumptive oxygen depletion^2,3^. In this case, oxygen levels higher than a typical cell incubator would be required to achieve physiological or cellular normoxia. The definition of pericellular oxygen concentrations is complex and has been reviewed in detail elsewhere^2^.

In hypoxic conditions, cells undergo adaptations to survive. The master regulator of the hypoxic response is the hypoxia inducible factor (HIF) pathway. Where, for example, HIF-1a is stabilised under hypoxic conditions, translocates to the nucleus and produces a hypoxic response^4^. As a result of these adaptations, cells cultured in hypoxic conditions have a distinct phenotype from those cultured under normoxic conditions. For example, in vivo, cancer cells often grow in cellular hypoxia which heightens their cancer-like phenotype^5^.

Extracellular vesicles (EVs) are a diverse group of membrane-bound droplets that are secreted from cells in response to a range of stimuli. EVs play a role in intercellular signalling and can influence cellular phenotype for example, by inducing a cancer-like phenotype and conditioning the premetastatic niche^6,7^. The content of EVs is actively packaged by cellular apparatus and consequently relates to the contents of the parent cell. Thus, as the phenotype of a cell changes in response to hypoxic conditions, so too changes the phenotype of EVs produced in cellular hypoxia.

Extracellular vesicles have accrued increasing research interest in recent years and the potential for EVs for therapy is highly anticipated. However, the methods for their production can be arduous, and large numbers of EVs are often required for their study and for therapies. Naturally, there have been several approaches to increase the yield of EVs from cell cultures. These include modulation of temperature, pH, oxidative stress, and notably, hypoxia^8^.

There are mixed reports in the literature addressing the effects of hypoxia on EV production and phenotype, however a consensus has not yet been reached. Most reports investigating EV number indicate that hypoxic conditions can induce EV production, but some reports maintain that there is little-to-no effect. Experiments examining the effects of hypoxia on EVs are often performed with cells exposed to acute hypoxia (AH), typically lasting 72 h or less^9^. We hypothesised that this duration was insufficient for full adaptation to hypoxic conditions and therefore skewed previous results towards non-fully adapted cells. Here, we characterise the effects of acute and chronic hypoxic (CH) culture on cells and the EVs they produce by generating hypoxic HepG2 and PC3 cells. By controlling the passage, cell number and medium volume and allowing a period of full adaptation to hypoxic conditions, the effects of hypoxia on cell behaviour and phenotype and on EV production were studied in isolation.

## Methods

Unless otherwise noted, all materials were obtained from Thermo Fisher Scientific (Vantaa, Finland).

### Production of EV-depleted foetal bovine serum

Foetal bovine serum was diluted with an equal volume of RPMI 1640 and centrifuged (110,000 × *g*, 15 h, 4 °C) in an Optima L80 XP ultracentrifuge with a SW 32 Ti rotor (k-factor 325) (both Beckman Coulter, CA, USA) to pellet bovine EVs. The supernatant was passed through a disposable 0.2 μm polyethersulphone filter and stored at -20 °C until further use.

### Cell culture

HepG2 (Sigma Aldrich, Espoo, Finland) and PC3 (European Collection of Authenticated Cell Cultures, Salisbury, UK) cells were grown in RPMI 1640 with 10% (v/v) EV-depleted FBS and 2 mM L-glutamine (henceforth, medium). During the experimental period, passage number, medium volume and culture area were equal between oxygen conditions. Cells were passaged every 72 h to maintain log phase growth. During passage, cells were washed twice with 1 × phosphate buffered saline without calcium or magnesium (PBS-) and then incubated at 37 °C until detachment was observed. The cells were counted, and 5 × 10^6^ cells were propagated into a new T175 flask in 25 mL medium.

**Fig. 1:**
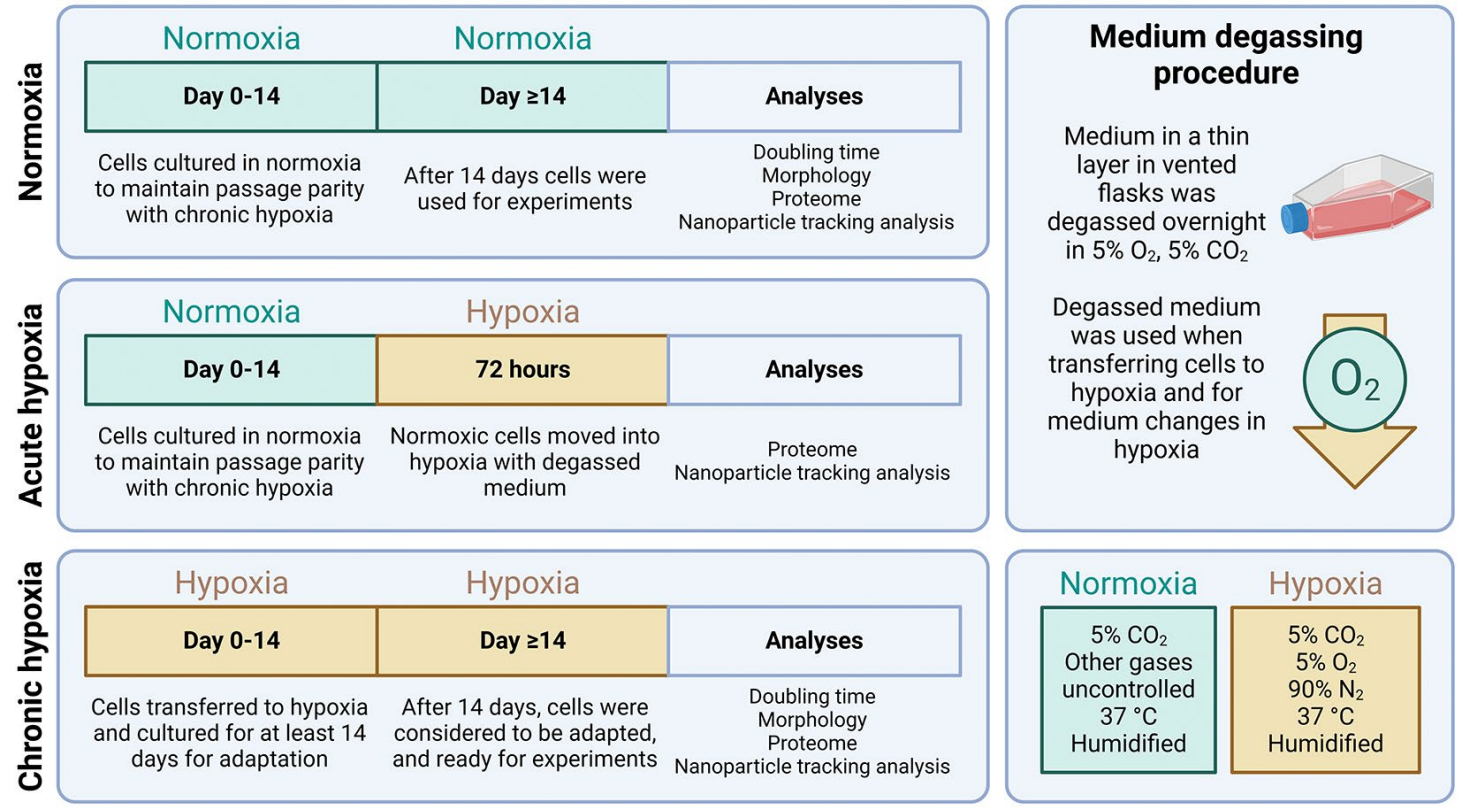
Experimental setup of hypoxia experiments. Three oxygen conditions were compared. In normoxia, cells were cultured in normoxic (5% CO_2_, other gases uncontrolled) conditions whilst maintaining passage number parity with chronic hypoxic conditions. In chronic hypoxia, cells were adapted to hypoxic conditions (5% O_2_, 5% CO_2_, 90% N_2_) for at least 14 days before experiments began. In acute hypoxia, cells were maintained in normoxic conditions until use, at which point they were transferred to hypoxic conditions for 72 h. When cells were transferred to hypoxic conditions, the medium was exchanged for degassed medium, which was also used for subsequent medium changes in hypoxia.

Cells were cultured under either normoxic conditions in a standard cell culture incubator (5% CO_2_, O_2_ uncontrolled), or in hypoxic conditions using a SCI-tive-N hypoxia workstation (Baker Ruskinn) (5% CO_2_, 5% O_2_, 90% N_2_). For CH experiments, cells were allowed an adaptation period of at least two weeks prior to experimentation. For AH experiments, cells from normoxic conditions were transferred into hypoxic conditions for 72 h, the duration of the experiment. In CH and AH experiments, medium was allowed to degas in 5% O_2_ hypoxic conditions overnight before use. These experimental setups are visualised in Fig. 1.

Conditioned medium containing EVs, was collected prior to passaging, and centrifuged (2,500 × *g*, 25 min, 4 °C) to remove detached cells and the supernatant was stored at -80 °C. At the end of the experiment, cells were lysed in assay-appropriate buffer and stored at -80 °C. Doubling time was calculated from the total number of cells at passage and the duration between passages. Brightfield micrographs of morphology were taken using an EVOS core microscope.

### Collection and purification of EVs

EVs were isolated from conditioned media by 0.2 μm polyethersulphone membrane filtration followed by centrifugation (110,000 × *g*, 2 h, 4 °C) in an Optima L80 XP ultracentrifuge with a SW 32 Ti rotor. Pellets were resuspended in 100 µL PBS- and stored at -80 °C until further use.

### Protein content quantification

Nanoparticle tracking analysisCA protein assay following the manufacturer’s instructions. Whole cells were lysed in radioimmunoprecipitation assay buffer before protein quantification. EVs were lysed as described in^10^. Briefly, EVs were incubated in lysis buffer (50mM Tris-HCl, 150mM NaCl, 1% triton X-100 (all Sigma-Aldrich), pH 8) for 30 min on ice and centrifuged at 17,000 × *g* for 20 min.

### Nanoparticle tracking analysis

Nanoparticle tracking analysis (NTA) was performed by the EV Core at the University of Helsinki. A PMX120 ZetaView (Particle Metrix, Germany) in scattered light mode with a laser wavelength of 488 nm (software version 8.05.16 SP3) was used to quantify particle size distribution and concentration.

### Proteomics

Protein samples were precipitated by addition of 100% trichloroacetic acid for 60 min, centrifugation at 20,000 × *g* for 30 min and were then washed three times in acetone. Proteins were reduced with 5 mM Tris(2-carboxyethyl)phosphine, alkylated with 10 mM iodoacetamide and trypsin digested for 16 h. Samples were acidified with 10% trifluoroacetic acid (TFA) and desalted using BioPureSPN PROTO 300 C18 Mini columns. Samples were dried and resuspended in 0.1% TFA, 1% acetonitrile in H_2_O and analysed using a timsTOF Pro 2 (Bruker) in data dependent mode.

In most analyses, the difference in mean spectral count between conditions was used to create a ranked list of detected proteins. The “Proteins with Values/Ranks” tool in StringDB version 11.5^11^ was used to generate a list of biological process (BP) GO terms which was then reduced by inputting the BP GO term and false discovery rates into Revigo version 1.8.1 using the *Homo sapiens* database and small output preset^12^. Chord plots were produced in R using the GOChord() function of the GOplot package with default parameters^13^.

For analysis of HepG2 EVs, the number of detected proteins was too small to use the “Proteins with Values/Ranks” tool. Therefore, a ranked list of proteins based on mean spectral count and expressed as fold change relative to normoxia was produced. Some contamination of bovine albumin was observed, presumably as residue from the medium and were excluded.

### Statistical tests

Statistical testing was performed using either Prism 10 (GraphPad Software, Boston, MA, USA) or RStudio, version 2023.12.1.402^14^. Data were analysed using either a one-way ANOVA and Tukey’s HSD post hoc test or with a two-tailed, unpaired Student’s t-test. Unless otherwise noted, when p-values are presented, they are calculated using a one-way ANOVA and Tukey’s HSD post hoc test. P-values ≤ 0.05 were considered significant. Hierarchical clustering, using the default “complete linkage” method, of mean spectral count was used to generate heatmaps in RStudio with the gplot package^15^.

## Results

### Chronic hypoxic culture does not affect cell morphology, cryopreservation capacity or doubling time

**Fig. 2:**
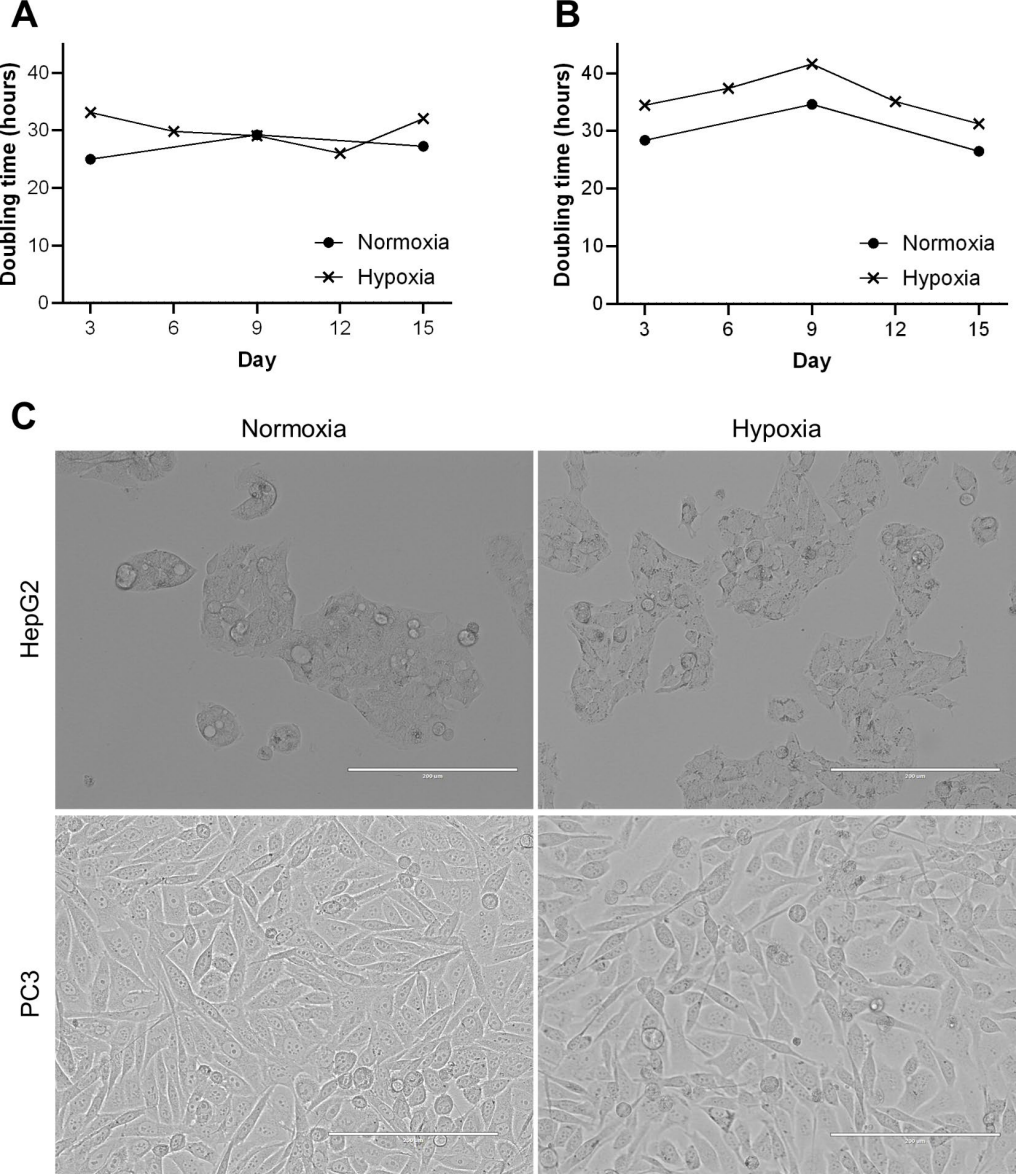
Doubling time and morphology in hypoxic culture** (A**,** B)**. Doubling time in hours, of HepG2 (A) and PC3 (B) cells when cultured in normoxia and 5% O_2_ hypoxia over a 15-day period after a period of adaptation for at least two weeks. Doubling time was calculated every three days in hypoxia and every six days in normoxia and was not significantly different between conditions (HepG2 *p* = 0.179, PC3 *p* = 0.080). **(C)** Brightfield micrographs of HepG2 (top) and PC3 (bottom) cells cultured in normoxia (left) and hypoxia (right). Scale bars are 200 μm. Hypoxia micrographs taken after culture in 5% O_2_ for approximately one month and after cryopreservation whilst maintaining hypoxic conditions.

For CH experiments, HepG2 and PC3 cells were cultured in hypoxic conditions for two weeks before experimentation. The doubling time of cells with matching passage number was calculated for both normoxia and CH and was unchanged (Student’s t-test, HepG2 *p* = 0.1768, PC3 *p* = 0.0804, *n* = 3) (Fig. 2A, B), although an insignificant trend towards increased doubling time in hypoxia for PC3 cells emerged (Fig. 2B). Brightfield images of cells under hypoxic conditions showed no gross morphological abnormalities compared with cells cultured in normoxic conditions (Fig. 2C). Cells in hypoxic conditions could be cryopreserved and recovered normally from cryogenic temperatures. The micrographs of hypoxic conditions in Fig. 2C show cells after cryopreservation and thawing in hypoxia.

### Effects of hypoxia on cell content

**Fig. 3:**
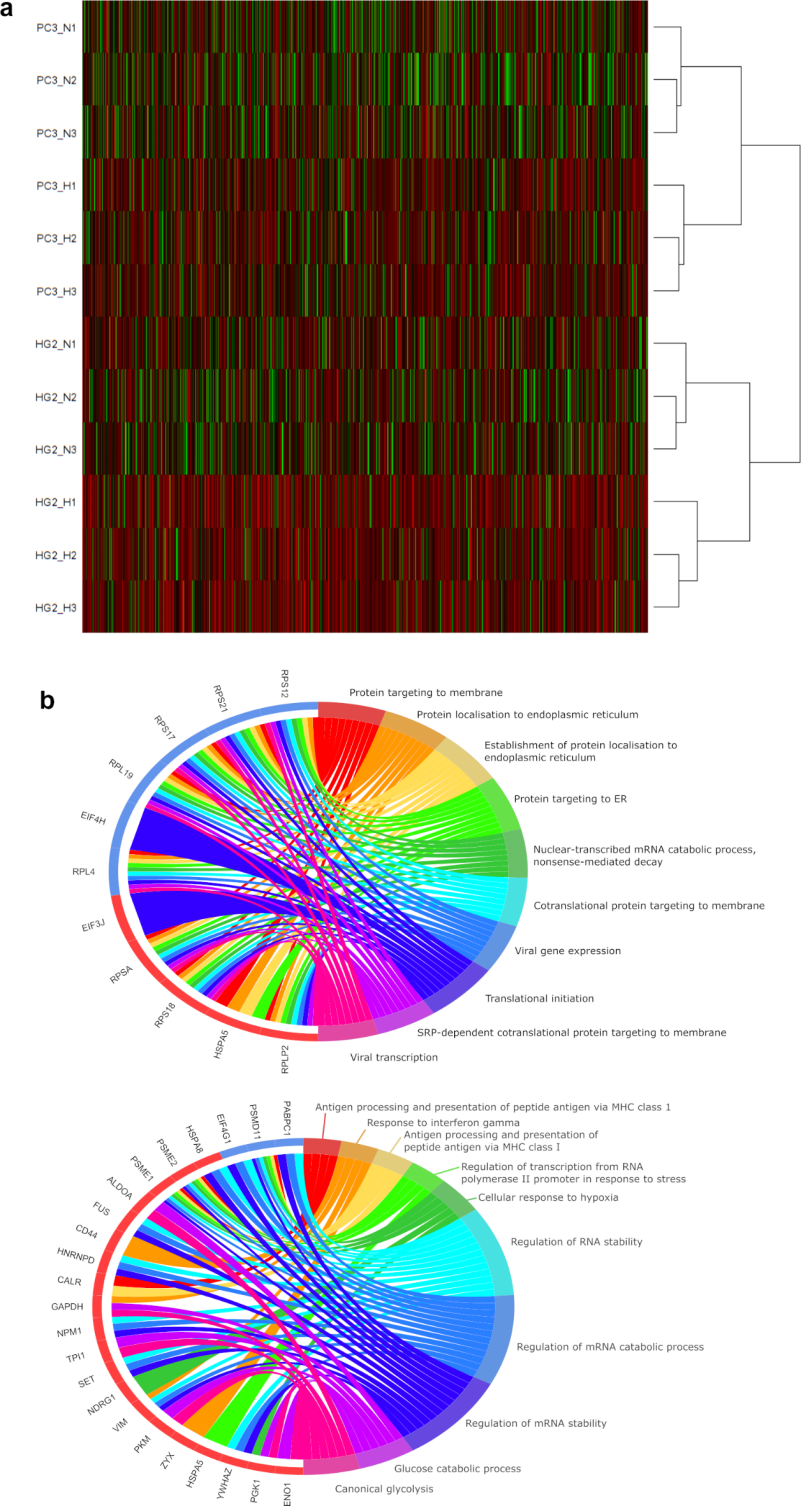
Phenotype of HepG2 and PC3 cells when cultured in chronic 5% O_2_ hypoxia.** (A)** Heatmap of protein expression in HepG2 and PC3 cells produced using the gplots package in Rstudio. Green denotes higher expression and red denotes lower expression. The dendrogram shows hierarchical clustering of samples. **(B**,** C)** Chord plots summarising the major changes in protein expression in HepG2 (B) and PC3 (C) cells produced from a list of BP GO terms produced using StringDB and the GOplot package in R. The right side displays the 10 Biological Process Gene Ontology terms most associated with the changes in protein expression. The left side shows the proteins with the greatest change in expression. A blue or red label denotes a downregulation or upregulation in conditions of hypoxia, respectively. The coloured chords denote membership of the highly changed genes within the most significant GO terms. *n* = 3. Abbreviations: AH = acute hypoxia, BP GO = biological process gene ontology, CH = chronic hypoxia, HG2 = HepG2, N = normoxia.

For whole cell analyses, a total of 4510 proteins were identified by proteomic analysis. In hierarchical clustering, both PC3 and HepG2 samples clustered by cell line and then by oxygen condition, as expected (Fig. 3A). Next, a ranked list of changed proteins between oxygen conditions was produced and processed in StringDB. A summary of the most significantly enriched BP GO terms were then visualised as chord plots (Fig. 3B, C). In these plots, the 10 most significantly changed proteins are displayed on the left side and connected to the most significantly associated BP GO terms.

There were 57 significantly enriched BP GO terms associated with the changes in protein expression between normoxia and chronic hypoxia in HepG2 cells and 28 in PC3 cells (Supplementary Table 1). These terms were summarised with Revigo, which produced a list of 28 terms associated with the changes in protein expression in HepG2 cells (Table 1) and 10 in PC3 cells (Table 2).

**Table 1:** Summarised list of biological process (BP) gene ontology (GO) terms associated with changes in protein expression in HepG2 cells cultured in chronic hypoxia. A list of BP GO terms was produced using StringDB version 11.5. The BP GO terms and their associated false discovery rates were then summarised using Revigo version 1.8.1 using the *Homo sapiens* database.

Biological process gene ontology term	Value	Frequency
Regulation of deoxyribonuclease activity	-2.143	0.056
Regulation of cellular amino acid metabolic process	-3.921	0.068
SRP-dependent co-translational protein targeting to membrane	-7.271	0.096
Regulation of hematopoietic stem cell differentiation	-2.292	0.096
Anaphase-promoting complex-dependent catabolic process	-2.523	0.124
Regulation of amine metabolic process	-4.07	0.180
Antigen processing and presentation of peptide antigen via MHC class I	-2.469	0.208
Nuclear-transcribed mRNA catabolic process, nonsense-mediated decay	-6.564	0.220
Regulation of DNA-templated transcription in response to stress	-2.009	0.220
Regulation of hematopoietic progenitor cell differentiation	-2.174	0.225
Wnt signalling pathway, planar cell polarity pathway	-4.099	0.231
Viral gene expression	-7.26	0.304
Tumour necrosis factor-mediated signalling pathway	-2.602	0.321
Regulation of morphogenesis of an epithelium	-2.367	0.360
Protein localisation to endoplasmic reticulum	-4.842	0.377
Translational initiation	-7.271	0.383
Regulation of stem cell differentiation	-2.523	0.445
ATP metabolic process	-2.114	0.670
Regulation of mRNA stability	-3.638	0.946
Protein modification by small protein removal	-2.921	0.946
RNA localisation	-2.174	0.991
Regulation of RNA splicing	-3.678	1.025
Protein-RNA complex assembly	-3.237	1.115
Protein-RNA complex organisation	-3.538	1.160
Response to decreased oxygen levels	-2.292	1.588
Regulation of apoptotic signalling pathway	-2.013	2.083
Generation of precursor metabolites and energy	-2.292	2.089
ncRNA processing	-2.022	2.337

The value and frequency values were calculated as part of the Revigo summary. Frequency indicates the proportion of the tested genome that makes up the listed GO term, lower values indicate more specific terms.

**Table 2:** Summarised list of biological process (BP) gene ontology (GO) terms associated with changes in protein expression in PC3 cells cultured in chronic hypoxia.

Biological process GO term	Value	Frequency
Regulation of transcription from RNA polymerase II promoter in response to hypoxia	-2.523	0.001
NADH metabolic process	-2.143	0.002
Canonical glycolysis	-3.959	0.002
Regulation of DNA-templated transcription in response to stress	-2.959	0.009
Response to type II interferon	-2.959	0.014
Regulation of RNA splicing	-2.319	0.052
Regulation of mRNA stability	-3.796	0.134
Establishment of protein localization to endoplasmic reticulum	-2.167	0.157
Translational initiation	-2.367	0.52
Protein folding	-2.444	1.059

A list of BP GO terms was produced using StringDB version 11.5, the BP GO terms and their associated false discovery rates were then summarised using Revigo version 1.8.1 using the *Homo sapiens* database. The value and frequency values were calculated as part of the Revigo summary. Frequency indicates the proportion of the tested genome that makes up the listed GO term, lower values indicate more specific terms.

### Effects of hypoxic culture on EV production

**Fig. 4:**
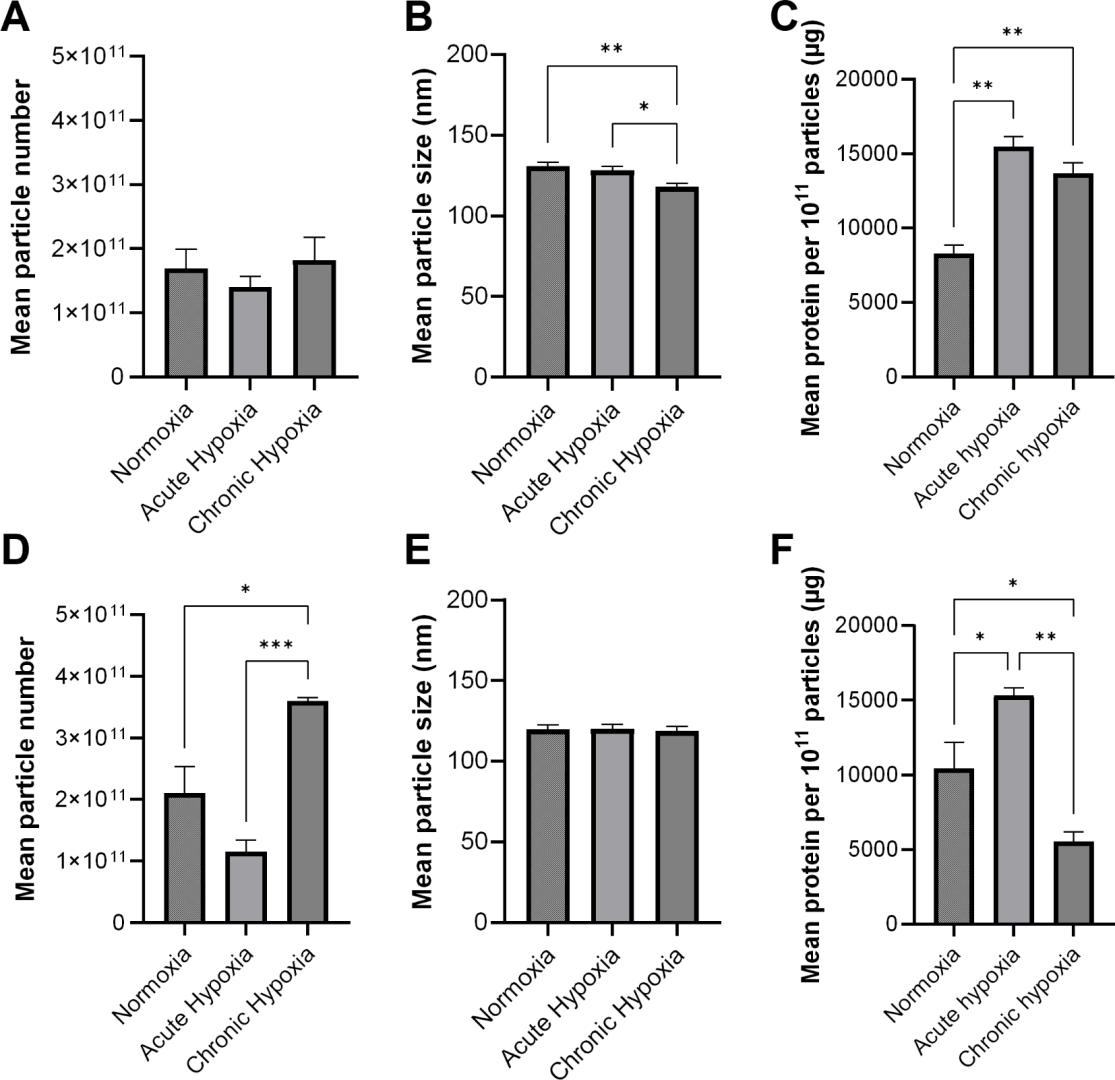
Extracellular vesicle production and content in different oxygen conditions. Panels **A**-**C **show values for HepG2, panels **D**-**F** show values for PC3. **(A, D)** Mean number of particles produced in 72 hours in different oxygen conditions normalised to cell number. **(B, E) **Mean size of particles produced in 72 hours in different oxygen conditions **(C, F)** Mean protein content of EVs produced in 72 hours in different oxygen conditions. Normoxia refers to culture under standard tissue culture conditions with atmospheric oxygen. Acute hypoxia refers to culture of cells in 5% O_2_ hypoxia for 72 hours. Chronic hypoxia also refers to culture of cells in 5% O_2_ with at least two weeks additional culture in 5% O_2_ hypoxia prior to experimentation. Error bars in all panels are SEM. Asterisks (*) denote significance determined by one-way ANOVA with Tukey’s HSD test, * = p ≤ 0.05, ** = p ≤ 0.01, *** = p ≤ 0.001.

The effects of hypoxic culture on EV production were probed by culturing PC3 and HepG2 cells in either normoxia, CH or AH. NTA was used to determine the number of particles produced in a 72-hour period. Other culture parameters e.g. cell number, passage and medium volume were constant between conditions. In HepG2 cells, the number of particles produced per cell was not significantly different between oxygen conditions (normoxia vs. AH *p* = 0.7761, normoxia vs. CH *p* = 0.9429, AH vs. CH *p* = 0.6057, Fig. 4A). In PC3 cells, there were significantly more particles per cell produced in CH than in normoxia or AH (*p* = 0.0150 and *p* = 0.0007, respectively, Fig. 4D). The number of particles produced in AH and normoxia were not significantly different (*p* = 0.0796, Fig. 4D).

In the same experimental setup, the mean particle size was determined by NTA. In HepG2, the mean particle size was approximately 118 nm in CH, 128 nm in AH and 130 nm in normoxia, which represents a significant decrease in CH versus both normoxia and AH (*p* = 0.0035 and *p* = 0.0336, respectively, Fig. 4B). In PC3, the mean particle size was approximately 118 nm in CH, 120 nm in AH and 119 nm in normoxia, which were not significantly different (Fig. 4E).

### Effects of hypoxia on EV content

The protein content of HepG2 and PC3 EVs from either AH, CH or normoxic conditions was calculated (Fig. 4C, F). In HepG2 EVs, the mean protein content was increased in both AH and CH relative to normoxia (*p* = 0.0010 and 0.0062 respectively, Fig. 4C). In PC3 EVs, the protein content was significantly different in each oxygen condition (normoxia vs. AH *p* = 0.0469, normoxia vs. CH *p* = 0.0468, AH vs. CH *p* = 0.0019, Fig. 4F). EVs from both cell lines and each oxygen condition were then subjected to proteomics analysis.

**Fig. 5:**
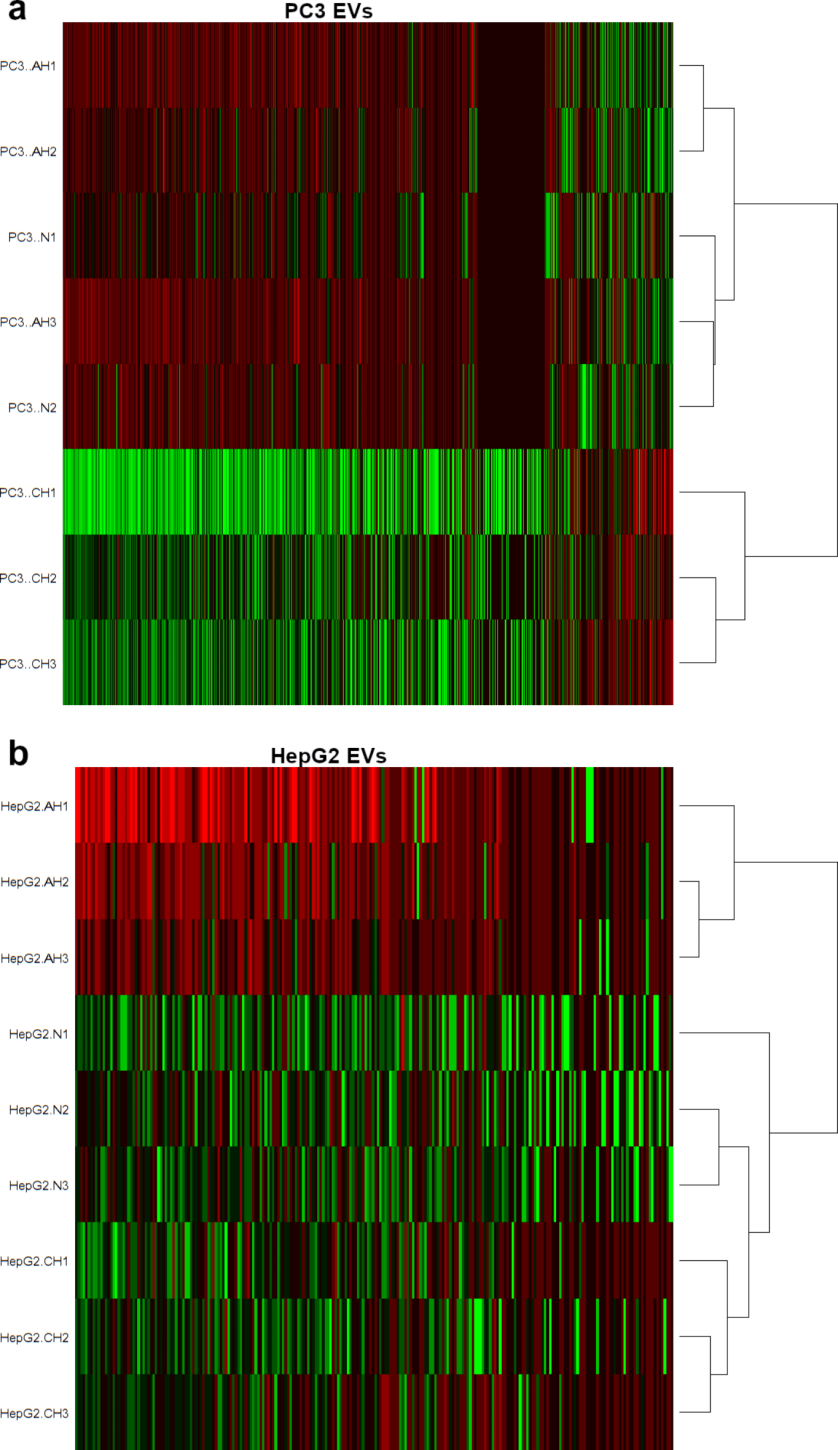
Protein expression in HepG2 and PC3 EVs in different oxygen conditions. Panel (**A**) shows data for HepG2, panel (**B**) shows data for PC3. The global protein expression of EVs was measured with proteomics and hierarchical clustering was performed in R using the gplots package. EVs were harvested for 72 h from cells cultured in either normoxic, AH, or CH conditions. Normoxia refers to culture under standard tissue culture conditions with atmospheric oxygen. AH refers to culture of cells in 5% O_2_ hypoxia for 72 h. CH refers to culture of cells in 5% O_2_ with at least two weeks additional culture in 5% O_2_ hypoxia prior to experimentation. *n* = 3, except in PC3 N where *n* = 2 Abbreviations: AH = acute hypoxia, CH = chronic hypoxia, N = normoxia.

In proteomic analysis, there were 1215 proteins detected in PC3 EVs, and 240 in HepG2 EVs. In hierarchical clustering, PC3 EVs from CH clustered separately to AH and normoxia, which clustered together (Fig. 5A). In HepG2 EVs, AH instead clustered separately from CH and normoxia (Fig. 5B), though these conditions did not cluster as closely together as AH and normoxia in PC3.

Similarly to the whole cell analysis, a reduced list of BP GO terms associated with the changes in protein expression in each comparison was produced with StringDB and Revigo. The reduced list of BP GO terms associated with the changes in PC3 EVs between each oxygen condition are shown in Table 3, the complete list of BP GO terms is shown in Supplementary Table 2. After reduction, there were 18 and 8 BP GO terms associated with the changes between CH and either normoxia or AH, respectively. All the terms associated with AH were redundant with CH. There was one term associated the changes between normoxia and AH which was also redundant with other changes.

**Table 3:** Summarised list of BP GO terms associated with changes in protein expression in PC3 EVs from cell cultured in different oxygen conditions.

Term ID	Name	Freq.	CH vs. *N*	AH vs. *N*	CH vs. AH
			Value	Value	Value
GO:0010608	Post-transcriptional regulation of gene expression	1.515	-2.387		-2.032
GO:0007010	Cytoskeleton organisation	0.862	-2.252		-2.143
GO:0007017	Microtubule-based process	0.835	-2.143		
GO:0022603	Regulation of anatomical structure morphogenesis	0.815	-2.036		
GO:0007155	Cell adhesion	0.706	-2.086		
GO:0030030	Cell projection organisation	0.661	-2.62		
GO:0120036	Plasma membrane bounded cell projection organisation	0.185	-2.495		-2.032
GO:0051130	Positive regulation of cellular component organisation	0.176	-2.26		
GO:0019058	Viral life cycle	0.131	-2.102		
GO:0032880	Regulation of protein localisation	0.124	-2.495		-2.143
GO:0030198	Extracellular matrix organisation	0.09	-3.252	-2.009	-3.62
GO:0008037	Cell recognition	0.087	-2.509		-2.62
GO:0001666	Response to hypoxia	0.034	-2.387		
GO:0007369	Gastrulation	0.033	-2.495		-2.004
GO:0010810	Regulation of cell-substrate adhesion	0.027	-2.036		-2.022
GO:0035567	Non-canonical Wnt signalling pathway	0.006	-2.237		
GO:0038093	Fc receptor signalling pathway	0.005	-2.102		
GO:0002576	Platelet degranulation	0.001	-2.387		

A summarised list of BP GO terms was produced using StringDB and Revigo. Value and frequency were calculated as part of the Revigo summary. Frequency indicates the proportion of the tested genome that makes up the listed GO term, lower values indicate more specific terms. Abbreviations: AH = acute hypoxia, CH = chronic hypoxia, N = normoxia.

There were too few proteins detected in HepG2 EVs to use StringDB as in other analyses. Instead, the complete list of detected proteins and their relative abundances are shown in Supplementary Table 3, and the 5 most upregulated and downregulated proteins from each condition are shown in Table 4. Despite the relatively small number of proteins detected, it was possible to discern differences between AH and CH based on these results. In Table 4, the five most positively and negatively regulated proteins in AH and CH are expressed as fold change versus normoxic EVs.

**Table 4:** Fold change in mean spectral count of the most highly changed proteins in HepG2 EVs in chronic or acute hypoxia relative to normoxia.

		Gene name	Uniprot name	Acute hypoxia	Chronic hypoxia
**Acute hypoxia**	**Most upregulated**	HAMP	HEPC_HUMAN	11.00	ND
		DNAH8	DYH8_HUMAN	2.67	2.33
		COL2A1	CO2A1_HUMAN	2.00	1.33
		ITIH3	ITIH3_HUMAN	2.00	1.00
		OGN	MIME_HUMAN	1.25	-2.00
	**Most downregulated**	PSMA5	PSA5_HUMAN	-17.00	-1.06
		GAPDH	G3P_HUMAN	-18.00	-1.16
		KRT9	K1C9_HUMAN	-25.00	1.48
		H1-2	H12_HUMAN	-29.67	-2.62
		FLNA	FLNA_HUMAN	-32.00	1.19
**Chronic hypoxia**	**Most upregulated**	ANG	ANGI_HUMAN	ND	10.00
		PCSK9	PCSK9_HUMAN	-7.00	4.57
		KRT2	K22E_HUMAN	1.00	4.00
		KRT5	K2C5_HUMAN	-1.33	3.75
		LTF	TRFL_HUMAN	ND	3.50
	**Most downregulated**	NPM1	NPM_HUMAN	ND	-7.00
		VASN	VASN_HUMAN	ND	-7.00
		RPS8	RS8_HUMAN	ND	-7.33
		RPL7	RL7_HUMAN	ND	-8.00
		H1-5	H15_HUMAN	ND	-11.00

The top set of 10 values are the 5 most positively and negatively regulated proteins in acute hypoxia, the bottom set of 10 values are the 5 most positively and negatively regulated proteins in chronic hypoxia. In both sets, the fold change of that protein in either chronic or acute hypoxia, relative to normoxia, are shown.

There was a trend towards decreased protein expression in hypoxic HepG2 EVs, which was more pronounced in AH. Of the 129 proteins detected in AH EVs, 104 were downregulated with a mean fold change of -4.92. Only 7 proteins were upregulated. Conversely, of the 192 proteins detected in CH EVs 103 were downregulated with a mean fold change in expression of -2.22. Seventy-four proteins were upregulated or unchanged. This is consistent with some of the protein changes in HepG2 cells, for example EIF4A3 was downregulated in AH an undetected in CH which likely indicates a decrease in transcription.

## Discussion

In this study, the effects of hypoxia on EV production and phenotype in cells cultured in AH and CH were examined. Most previous studies combining hypoxia and EVs have used short durations of hypoxia for cell adaptation, typically 72 h or less. It was hypothesised that adaptation to a new oxygen regime would continue for a longer period, which would disproportionately capture the early period of adaptation to the new oxygen regime in these studies and thereby skew the results. The number, size, and content of EVs secreted under AH, CH and normoxic conditions from PC3 and HepG2 cells in a tightly controlled environment were quantified. PC3 and HepG2 cells were chosen based on their differing metabolic oxygen requirements, specifically the propensity for HepG2 cells to induce metabolic oxygen depletion. PC3 and HepG2 are also examples of metastatic and non-metastatic cancer cell lines, respectively. However, since a non-malignant cell line was not included as control, it is not appropriate to draw conclusions regarding the effects of metastasis on hypoxic EV phenotype.

Given the heterogeneity of reported experimental procedures, possibly arising from the frequent paucity of reported experimental details, disagreement regarding the effects on hypoxia found in the literature are unsurprising. Literature reports suggest that hypoxic conditions induce EV production and alter EV composition (recently reviewed by Yaghoubi et al.^8^), the results herein support and refine this concept, as PC3 cells increased production of EVs only in CH. These findings also add nuance to the understanding of EV secretion in hypoxia. There are some reports of little-to-no effect of hypoxia on EV production similar to the observations of HepG2 cells in this study. For example, Ramteke et al. observed no increase in EV production after 72 h of 1% hypoxia in prostate cells^16^. The results presented herein suggest that an increase in EV production may have been observed if hypoxia had been maintained for a longer period. Moreover, we observed no increase in EV production in HepG2 cells in response to hypoxic conditions.

The response to hypoxia is primarily driven by HIFs 1 and 2 which are stabilised under hypoxic conditions. HIF1 mediates the response to acute or intense periods of hypoxia, and peaks in expression within hours of hypoxic insult^17^. Conversely, HIF2 mediates the response to chronic and less intense hypoxia and continues to increase in expression to 72 h^17^. Bister et al. showed that most EV experiments including hypoxia used an experimental period of 72 h or less of hypoxia^9^. This initially seems reasonable given the response times of HIF isoforms, however we hypothesised that despite the peak of HIF expression, adaptation to hypoxic conditions would continue for longer than 72 h.

In this study, it was shown that cell lines respond to hypoxia in isolation over a longer period than is most commonly used by studies combining hypoxia and EVs. The changes in production and composition of EVs in response to hypoxic conditions varied based on cell line. It is noteworthy that in the present study HepG2 and PC3 cells exhibited the greatest change in protein expression after different durations of hypoxia.

### Effects on cells

These results confirm that the effects of hypoxia on the cells were detectable and in agreement with those that might be expected based on literature sources. Although HIF1 and HIF2 were not detected, the effects of a hypoxic response were apparent.

The BP GO terms associated with the changes in HepG2 cells in CH suggest that they underwent metabolic rearrangements when cultured in hypoxia as would be expected for cells in hypoxic conditions (Table 1). Specifically, the term “Response to decreased oxygen levels” was associated, as expected for cells in hypoxic conditions. Furthermore, BP GO terms indicating alterations in protein synthesis and targeting the endoplasmic reticulum and membrane were associated. This likely represents activation of the ER stress response, as a cytoprotective adaptive mechanism to hypoxic conditions^18^. The Wnt and tumour necrosis factor pathways were also regulated. Both pathways have been implicated in the accumulation of HIF1a on the RNA level and protein level respectively^19,20^.

In PC3, the enriched BP GO terms were associated with metabolic alterations that would be expected in hypoxic conditions, e.g. “Canonical glycolysis” and “NADH metabolic process” (Table 2). In addition, there were also terms associated with similar changes observed in HepG2 cells, such as protein localisation to the endoplasmic reticulum. Other expected terms for cells in hypoxic conditions such as “regulation of transcription from RNA polymerase II promoter in response to hypoxia” were associated.

### Effects on EVs

The contents of the EVs for both cell lines were consistent with their putative tissue of origin. In PC3 EVs, the proteins detected were consistent with prostatic cells. When compared to a list of 1935 prostate enriched proteins from the Human Protein Atlas, 185 were also detected in PC3 EVs^21^. Furthermore, from a list of 180 proteins identified as being uniquely expressed in PC3 cells compared to LNCaP cells, 40 were detected in PC3 EVs^22^. Notably, the prostate markers KLK3 (prostate specific antigen) and AR (androgen receptor) were not detected, consistent with literature reports that PC3 cells do not express either protein^23^.

The changes in PC3 EVs in CH were associated with response to hypoxia, as expected. In addition, the Wnt signalling pathway was regulated, which has been implicated in hypoxic response. Furthermore, there were changes related to modification and interactions with the extracellular matrix indicated by BP GO terms including “cytoskeletal rearrangements”, “cell adhesion” and “extracellular matrix organisation”. Interestingly, the BP GO term, “gastrulation” was associated with these changes, whilst PC3 cells clearly do not undergo gastrulation, this may be indicative of other related processes, such as epithelial-to-mesenchymal transition^24^. Combined, these results may indicate a more invasive phenotype in PC3 cells in hypoxic conditions.

In HepG2 EVs, the proteins detected were consistent with cells of hepatic origin. Of the 10 proteins with highest combined spectral count, 8 were liver-specific or enriched proteins i.e. fibronectin, alpha-2-macroglobulin, albumin, reelin, alpha-fetoprotein, fetuin A, serotransferrin, complement C4-A. The remaining top 10 proteins were beta actin, which is ubiquitously expressed in most tissues and haemoglobin. Whilst haemoglobin is not synthesised in hepatocytes under normal conditions its expression can be induced in CH and in non-alcoholic steatohepatitis^25,26^. There is no overlap in the top 5 positively and negatively regulated proteins (Table 4), which indicates a response to hypoxic conditions even though HepG2 cells induce consumptive oxygen depletion in experimental normoxia^3,27^.

Several differentially expressed proteins are particularly noteworthy and indicative of a hypoxic response that changes over time. Filamin A is the most downregulated protein in HepG2 EVs cultured in AH. Cleavage of Filamin A is part of the hypoxic response^28^, where, under hypoxic conditions, it is cleaved by calpain and binds and activates HIF1a. It is probable that depletion of filamin A in AH, but not in CH reflects a transient activation of HIF1a by this mechanism.

Similarly, hepcidin is upregulated 11-fold in AH but was undetected in CH. Hepcidin is a central regulator of iron homeostasis that inhibits iron export from cells^29^. It is negatively regulated by the HIF system^30^ and thus the downregulation observed in CH conditions is expected. However, upregulation in AH is unexpected as previous reports have shown that mRNA expression of hepcidin in HepG2 cells decreased over 48 h of hypoxia^31^. These findings suggest that hepcidin may be exported in EVs in response to hypoxia or that production increases acutely before decreasing in chronic hypoxia.

Keratin 9 also showed a biphasic response to hypoxia, it was strongly downregulated in AH EVs but slightly upregulated in CH. Regulation of keratin 9 in EVs in response to AH has been reported previously in mesenchymal stem cells^7,32^. Hypoxia has also been reported to induce disassembly of cytokeratin filaments in A549 cells^33^.

PSMA5 was strongly downregulated in AH EVs but not in CH EVs. PSMA5 is part of the 26 S proteasome that is responsible for the degradation of HIF1a, and therefore its transient downregulation may indicate upregulation of HIF1a in response to AH. PSMA5 downregulation in hypoxia has been reported elsewhere^34^.

The biphasic phenotype of HepG2 EVs between chronic and AH is further demonstrated by the most upregulated proteins in CH EVs. Angiogenin, a key regulator of angiogenesis that is controlled by the HIF pathway is the most upregulated protein (11-fold change) in CH but was not detected in AH. Furthermore, PCSK9, which is implicated in survival of peripheral vascular networks, was the second most upregulated protein in CH but was downregulated in AH.

### Metabolic oxygen depletion

The metabolic activity of HepG2 cells induces consumptive oxygen depletion in normoxic culture^27,35^. That is, in experimental normoxia, HepG2 cells produce a state of cellular hypoxia prior to the application of experimental hypoxia. These data suggest that since HepG2 cells experience cellular hypoxia under normoxic conditions, their capacity to respond to experimental hypoxia, in terms of increased EV production is limited, at least with the degree of hypoxia used in this study. The expression profile of HepG2 EVs was altered in AH before returning to a profile more similar to experimental normoxia in CH, however, the number of EVs produced was unaffected. Although the number of EVs produced by HepG2 cells was unaffected by hypoxia, there was significantly more protein per EV in both AH and CH. These results, in combination with the altered EV proteome in AH, suggest that capacity remains for further adaptation to hypoxia even after consumptive oxygen depletion.

Conversely, we are not aware of similar reports of consumptive oxygen depletion in PC3 cells, which are likely in cellular normoxia in experimental normoxia conditions. This allows PC3 cells to adapt more obviously to experimental hypoxia. In PC3 EVs, the largest adaptations in the proteome and EV production occurred only after CH. Since other factors were constant, it is probable that differing oxygen demand of HepG2 and PC3 cells is responsible for the discrepancy in behaviour, though further experimentation is required to show this definitively. We hypothesise that cells which do not increase EV production in response to hypoxic conditions may fail to do so due to consumptive oxygen depletion.

These results, particularly in PC3, demonstrate that there is an adaptive response to hypoxia in EVs that takes longer than 72 h to establish. Whilst there is commonality between EVs arising from cells cultured under AH conditions and those from CH, the commonality is mainly one-directional, indicating a continuing process of adaptation.

While the EVs arising from the cancer cell types used in this study are unlikely to be therapeutically useful, the broader concepts discussed may be impactful on the manufacture of therapeutic EVs and for studying physiological events in vitro. Specifically, the number of EVs required for therapies is large, and therefore, the use of hypoxia to increase the yield of EVs from cultured cells is potentially valuable. These results add nuance to the understanding of when hypoxia is likely to be an appropriate tool for this purpose, i.e. in cells that do not induce consumptive oxygen depletion. Furthermore, the time taken for EVs to adapt to hypoxia is longer than has previously been reported and thus therapeutic EVs produced during this adaptation period may exhibit a variable phenotype. Future studies should investigate if the phenotype of EVs continues to adapt to hypoxia for periods longer than those tested herein and identify the precise time required for the phenotype of EVs to stabilise.

Cells could be cultured simply in low oxygen conditions long term without affecting morphology or doubling time. AH culture did not lead to an increase in EV production in either HepG2 or PC3 cells. In fact, there was a non-significant decrease in the mean number of particles produced in both cell lines. However, after CH, the number of particles was significantly increased in PC3 cells. This further indicates a process of continuing adaptation to hypoxia. The reduced effect in HepG2 cells is likely a result of consumptive oxygen depletion in normoxic conditions^35^. The mean size of EVs was unchanged in PC3 cells and changed only slightly in HepG2 cells, although this change was statistically significant, the biological significance is dubious. The magnitude of change was a few nanometres and any of the three major types of EVs (exosomes, 30–150 nm; microvesicles, 100–1000 nm; and apoptotic bodies, 50–5000 nm) could occupy this size category^36^.

## Conclusions

Adaptation to hypoxia lasting longer than 72 h can be observed in EVs derived from HepG2 and PC3 cells. In PC3 cells, the greatest changes in protein expression and EV production were observed only after CH. In HepG2 cells, EV production did not change in response to hypoxia, the greatest changes in protein expression were observed after AH. The varied response in EVs is likely a result of varying metabolic oxygen demand for each cell type. CH more closely recapitulates a hypoxic niche than acute hypoxic conditions and is therefore likely a more physiologically relevant culture modality. Crucially, these results indicate that the most commonly used durations for hypoxia experiments with EVs are insufficient for complete adaptation and may alter the results of such experiments. Therefore, the duration of hypoxia should be considered in more detail in the design of future studies.

## Electronic supplementary material

Below is the link to the electronic supplementary material.

Supplementary Material 1

Supplementary Material 2

Supplementary Material 3

## Data Availability

All unprocessed data is available from the corresponding author upon reasonable request.
