# Supplementary material for: Chronic hypoxia for the adaptation of extracellular vesicle phenotype
Source: Sci Rep. 2024 Oct 24;14:25189. doi: 10.1038/s41598-024-73453-1 (PMC11502752; doi:10.1038/s41598-024-73453-1)
Supplement: Supplementary file 1 — Supplementary Material 1 [file 41598_2024_73453_MOESM1_ESM.docx]

**Supplementary Table 1** – Complete list of biological process gene ontology terms associated with the changes in HepG2 and PC3 cells when cultured in either normoxia or chronic 5% hypoxia. Terms and enrichment score predicted using StringDB version 11.5.

| **HepG2** | | |
| --- | --- | --- |
| **#term ID** | **term description** | **enrichment score** |
| GO:0006413 | Translational initiation | 0.284625 |
| GO:0006614 | SRP-dependent co-translational protein targeting to membrane | 0.348786 |
| GO:0019083 | Viral transcription | 0.379885 |
| GO:0019080 | Viral gene expression | 0.364129 |
| GO:0006613 | Co-translational protein targeting to membrane | 0.321389 |
| GO:0000184 | Nuclear-transcribed mRNA catabolic process, nonsense-mediated decay | 0.137513 |
| GO:0045047 | Protein targeting to er | 0.23862 |
| GO:0072599 | Establishment of protein localisation to endoplasmic reticulum | 0.23492 |
| GO:0070972 | Protein localisation to endoplasmic reticulum | 0.20406 |
| GO:0006612 | Protein targeting to membrane | 0.133555 |
| GO:0000956 | Nuclear-transcribed mRNA catabolic process | 0.268971 |
| GO:0046395 | Carboxylic acid catabolic process | 0.165686 |
| GO:0060071 | Wnt signalling pathway, planar cell polarity pathway | 0.219815 |
| GO:0033238 | Regulation of cellular amine metabolic process | 0.220989 |
| GO:0006521 | Regulation of cellular amino acid metabolic process | 0.232403 |
| GO:0043484 | Regulation of RNA splicing | 0.23923 |
| GO:0006402 | mRNA catabolic process | 0.204568 |
| GO:0035567 | Non-canonical wnt signalling pathway | 0.18411 |
| GO:0043488 | Regulation of mRNA stability | 0.288935 |
| GO:0071826 | Ribonucleoprotein complex subunit organisation | 0.112245 |
| GO:0006401 | RNA catabolic process | 0.21007 |
| GO:0022618 | Ribonucleoprotein complex assembly | 0.100597 |
| GO:0048024 | Regulation of mRNA splicing, via spliceosome | 0.165655 |
| GO:0090175 | Regulation of establishment of planar polarity | 0.194506 |
| GO:0061013 | Regulation of mRNA catabolic process | 0.261207 |
| GO:0070646 | Protein modification by small protein removal | 0.140602 |
| GO:0042254 | Ribosome biogenesis | 0.242001 |
| GO:0043487 | Regulation of RNA stability | 0.275708 |
| GO:0090150 | Establishment of protein localisation to membrane | 0.092213 |
| GO:0072329 | Monocarboxylic acid catabolic process | 0.169868 |
| GO:0050684 | Regulation of mRNA processing | 0.124864 |
| GO:0033209 | Tumour necrosis factor-mediated signalling pathway | 0.267565 |
| GO:0031145 | Anaphase-promoting complex-dependent catabolic process | 0.145227 |
| GO:0061418 | Regulation of transcription from RNA polymerase II promoter in response to hypoxia | 0.152573 |
| GO:2000736 | Regulation of stem cell differentiation | 0.20431 |
| GO:0002474 | Antigen processing and presentation of peptide antigen via MHC class I | 0.087598 |
| GO:1905330 | Regulation of morphogenesis of an epithelium | 0.169814 |
| GO:0006091 | Generation of precursor metabolites and energy | 0.059475 |
| GO:0036293 | Response to decreased oxygen levels | 0.124941 |
| GO:1902036 | Regulation of hematopoietic stem cell differentiation | 0.212674 |
| GO:0006403 | RNA localisation | 0.196884 |
| GO:0006631 | Fatty acid metabolic process | 0.207878 |
| GO:0016579 | Protein deubiquitination | 0.147218 |
| GO:0042255 | Ribosome assembly | 0.574298 |
| GO:1901532 | Regulation of hematopoietic progenitor cell differentiation | 0.19512 |
| GO:0019395 | Fatty acid oxidation | 0.198085 |
| GO:0019884 | Antigen processing and presentation of exogenous antigen | 0.1316 |
| GO:0042590 | Antigen processing and presentation of exogenous peptide antigen via MHC class I | 0.094471 |
| GO:1903312 | Negative regulation of mRNA metabolic process | 0.183723 |
| GO:0032070 | Regulation of deoxyribonuclease activity | 2.57738 |
| GO:0046034 | ATP metabolic process | 0.110265 |
| GO:0044282 | Small molecule catabolic process | 0.124209 |
| GO:0070482 | Response to oxygen levels | 0.112973 |
| GO:0034470 | ncRNA processing | 0.153926 |
| GO:2001233 | Regulation of apoptotic signalling pathway | 0.11711 |
| GO:0043620 | Regulation of DNA-templated transcription in response to stress | 0.160623 |
| GO:0002478 | Antigen processing and presentation of exogenous peptide antigen | 0.132272 |
| PC3 | | |
| **#term ID** | **term description** | **enrichment score** |
| GO:0006007 | Glucose catabolic process | 1.67732 |
| GO:0061621 | Canonical glycolysis | 1.77043 |
| GO:0043488 | Regulation of mRNA stability | 0.32814 |
| GO:0061013 | Regulation of mRNA catabolic process | 0.300868 |
| GO:0043487 | Regulation of RNA stability | 0.311817 |
| GO:0002474 | Antigen processing and presentation of peptide antigen via MHC class I | 0.214181 |
| GO:0043618 | Regulation of transcription from RNA polymerase II promoter in response to stress | 0.251908 |
| GO:0071456 | Cellular response to hypoxia | 0.283728 |
| GO:0002479 | Antigen processing and presentation of exogenous peptide antigen via MHC class I, tap-dependent | 0.239452 |
| GO:0034341 | Response to interferon-gamma | 0.35379 |
| GO:0043620 | Regulation of DNA-templated transcription in response to stress | 0.24021 |
| GO:0006096 | Glycolytic process | 1.60168 |
| GO:0060333 | Interferon-gamma-mediated signalling pathway | 0.324316 |
| GO:0036294 | Cellular response to decreased oxygen levels | 0.260828 |
| GO:0001666 | Response to hypoxia | 0.243731 |
| GO:0042590 | Antigen processing and presentation of exogenous peptide antigen via MHC class I | 0.22434 |
| GO:0061418 | Regulation of transcription from RNA polymerase II promoter in response to hypoxia | 0.21683 |
| GO:0006457 | Protein folding | 0.165544 |
| GO:0071346 | Cellular response to interferon-gamma | 0.393269 |
| GO:0019221 | Cytokine-mediated signalling pathway | 0.119622 |
| GO:0006413 | Translational initiation | 0.444137 |
| GO:0006165 | Nucleoside diphosphate phosphorylation | 1.17804 |
| GO:0043484 | Regulation of RNA splicing | 0.285614 |
| GO:0036293 | Response to decreased oxygen levels | 0.227138 |
| GO:0046939 | Nucleotide phosphorylation | 1.14048 |
| GO:0072599 | Establishment of protein localisation to endoplasmic reticulum | 0.1145 |
| GO:0002253 | Activation of immune response | 0.080885 |
| GO:0006734 | NADH metabolic process | 1.34554 |
