# Supplementary material for: Chronic hypoxia for the adaptation of extracellular vesicle phenotype
Source: Sci Rep. 2024 Oct 24;14:25189. doi: 10.1038/s41598-024-73453-1 (PMC11502752; doi:10.1038/s41598-024-73453-1)
Supplement: Supplementary file 2 — Supplementary Material 2 [file 41598_2024_73453_MOESM2_ESM.docx]

**Supplementary Table 2** - Complete list of biological process gene ontology terms associated with the changes in protein expression in PC3 EVs when cultured in either normoxia, acute hypoxia or chronic hypoxia. Terms, enrichment score and FDR predicted using StringDB version 11.5. Abbreviations: FDR = false discovery rate, CH = chronic hypoxia, N = normoxia, AH = acute hypoxia

| **#term ID** | **term description** | **CH vs. N** | | **CH vs. AH** | | **AH vs. N** | |
| --- | --- | --- | --- | --- | --- | --- | --- |
|  |  | **Enrichment score** | **FDR** | **Enrichment score** | **FDR** | **Enrichment score** | **FDR** |
| GO:0030198 | Extracellular matrix organisation | 0.322078 | 0.00056 | 0.337831 | 0.00024 | 0.347785 | 0.0098 |
| GO:0030030 | Cell projection organisation | 0.110874 | 0.0024 |  |  |  |  |
| GO:0120036 | Plasma membrane bounded cell projection organisation | 0.111537 | 0.0024 |  |  |  |  |
| GO:0008037 | Cell recognition | 0.120088 | 0.0031 | 0.165147 | 0.0024 |  |  |
| GO:0007369 | Gastrulation | 0.4784 | 0.0032 | 0.444107 | 0.0099 |  |  |
| GO:0032880 | Regulation of protein localisation | 0.048701 | 0.0032 | 0.069904 | 0.0072 |  |  |
| GO:0120031 | Plasma membrane bounded cell projection assembly | 0.176939 | 0.0032 | 0.223332 | 0.0093 |  |  |
| GO:1903829 | Positive regulation of protein localisation | 0.062793 | 0.0032 | 0.080987 | 0.0072 |  |  |
| GO:0001666 | Response to hypoxia | 0.027651 | 0.0041 |  |  |  |  |
| GO:0002576 | Platelet degranulation | 0.333667 | 0.0041 |  |  |  |  |
| GO:0010608 | Posttranscriptional regulation of gene expression | 0.006599 | 0.0041 | 0.010621 | 0.0093 |  |  |
| GO:0070925 | Organelle assembly | 0.073348 | 0.0041 |  |  |  |  |
| GO:0022610 | Biological adhesion | 0.132016 | 0.005 | 0.136186 | 0.0099 |  |  |
| GO:0030031 | Cell projection assembly | 0.160606 | 0.005 |  |  |  |  |
| GO:0060341 | Regulation of cellular localisation | 0.051842 | 0.005 |  |  |  |  |
| GO:1904951 | Positive regulation of establishment of protein localisation | 0.051926 | 0.005 | 0.070659 | 0.0099 |  |  |
| GO:0036293 | Response to decreased oxygen levels | 0.02552 | 0.0051 |  |  |  |  |
| GO:0048468 | Cell development | 0.123404 | 0.0055 |  |  |  |  |
| GO:0051130 | Positive regulation of cellular component organisation | 0.079081 | 0.0055 |  |  |  |  |
| GO:0000226 | Microtubule cytoskeleton organisation | 0.19251 | 0.0056 |  |  |  |  |
| GO:0007010 | Cytoskeleton organisation | 0.126095 | 0.0056 | 0.154475 | 0.0072 |  |  |
| GO:0035567 | Non-canonical Wnt signalling pathway | 0.016536 | 0.0058 |  |  |  |  |
| GO:0007017 | Microtubule-based process | 0.12159 | 0.0072 |  |  |  |  |
| GO:0019058 | Viral life cycle | 0.0679 | 0.0079 |  |  |  |  |
| GO:0038093 | Fc receptor signalling pathway | 0.023362 | 0.0079 |  |  |  |  |
| GO:0042327 | Positive regulation of phosphorylation | 0.00707 | 0.0079 |  |  |  |  |
| GO:0007155 | Cell adhesion | 0.132807 | 0.0082 |  |  |  |  |
| GO:0001704 | Formation of primary germ layer | 0.512817 | 0.0092 |  |  |  |  |
| GO:0010810 | Regulation of cell-substrate adhesion | 0.24778 | 0.0092 | 0.233622 | 0.0095 |  |  |
| GO:0022603 | Regulation of anatomical structure morphogenesis | 0.123061 | 0.0092 |  |  |  |  |
| GO:0030099 | Myeloid cell differentiation | 0.233257 | 0.0092 |  |  |  |  |
| GO:0045937 | Positive regulation of phosphate metabolic process | 0.007645 | 0.0092 |  |  |  |  |
| GO:1903827 | Regulation of cellular protein localisation | 0.064062 | 0.0092 |  |  |  |  |
| GO:0033043 | Regulation of organelle organisation | 0.010519 | 0.0094 |  |  |  |  |
| GO:0030097 | Hemopoiesis | 0.159546 | 0.0099 |  |  |  |  |
| GO:0001934 | Positive regulation of protein phosphorylation | 0.132922 | 0.01 |  |  |  |  |
