# Supplementary material for: Chronic hypoxia for the adaptation of extracellular vesicle phenotype
Source: Sci Rep. 2024 Oct 24;14:25189. doi: 10.1038/s41598-024-73453-1 (PMC11502752; doi:10.1038/s41598-024-73453-1)
Supplement: Supplementary file 3 — Supplementary Material 3 [file 41598_2024_73453_MOESM3_ESM.docx]

**Supplementary Table 3** – list of proteins detected in HepG2 EVs from different oxygen conditions. Values are mean spectral count for each protein (n=3) and associated SEM. Abbreviations: SEM = standard error of the mean

| **Gene name** | **Description** | **Chronic hypox.** | | **Normoxia** | | **Acute hypoxia** | |
| --- | --- | --- | --- | --- | --- | --- | --- |
|  |  | **Mean** | **SEM** | **Mean** | **SEM** | **Mean** | **SEM** |
| FN1 | Fibronectin | 146.33 | 2.46 | 136.33 | 10.02 | 44.17 | 16.00 |
| A2M | Alpha-2-macroglobulin | 52.33 | 9.87 | 68.00 | 23.30 | 11.00 | 2.29 |
| ALB | Albumin | 66.50 | 10.90 | 44.00 | 12.98 | 14.67 | 1.36 |
| RELN | Reelin | 42.00 | 1.89 | 64.33 | 8.85 | 12.33 | 5.26 |
| AFP | Alpha-fetoprotein | 52.50 | 1.61 | 44.67 | 3.06 | 15.50 | 2.93 |
| AHSG | Alpha-2-HS-glycoprotein | 54.83 | 1.59 | 36.17 | 3.00 | 20.50 | 5.80 |
| ACTB | Actin, cytoplasmic 1 | 37.17 | 3.42 | 37.50 | 0.58 | 16.00 | 1.32 |
| TF | Serotransferrin | 38.17 | 5.78 | 27.33 | 1.92 | 13.33 | 4.15 |
| HBA2 | Hemoglobin subunit alpha | 27.67 | 0.73 | 26.67 | 1.74 | 17.67 | 3.63 |
| C4A | Complement C4-A | 23.33 | 2.13 | 23.33 | 2.77 | 5.33 | 2.80 |
| C3 | Complement C3 | 18.17 | 1.59 | 23.00 | 5.41 | 5.50 | 1.15 |
| LYZ | Lysozyme C | 17.17 | 1.09 | 18.50 | 4.04 | 8.83 | 4.17 |
| TLN1 | Talin-1 | 16.33 | 0.44 | 20.50 | 4.16 | 6.17 | 1.59 |
| APOE | Apolipoprotein E | 16.00 | 2.18 | 18.00 | 3.40 | 6.50 | 1.15 |
| TUBA4A | Tubulin alpha-4A chain | 10.17 | 1.45 | 13.50 | 0.87 | 8.17 | 2.32 |
| ITIH2 | Inter-alpha-trypsin inhibitor heavy chain H2 | 10.00 | 0.76 | 12.00 | 2.50 | 5.33 | 0.73 |
| FGA | Fibrinogen alpha chain | 13.50 | 1.53 | 10.17 | 0.44 | 2.00 | 1.04 |
| FBLN1 | Fibulin-1 | 12.67 | 1.42 | 10.00 | 0.87 | 2.67 | 0.33 |
| APOA1 | Apolipoprotein A-I | 12.00 | 0.76 | 10.83 | 0.73 | 1.83 | 0.44 |
| THBS1 | Thrombospondin-1 | 8.00 | 0.76 | 12.67 | 0.17 | 2.67 | 0.93 |
| IGFBP1 | Insulin-like growth factor-binding protein 1 | 14.33 | 6.21 | 5.67 | 5.42 | 2.67 | 1.45 |
| GC | Vitamin D-binding protein | 9.00 | 0.50 | 8.83 | 0.17 | 4.50 | 0.76 |
| H1-2 | Histone H1.2 | 5.67 | 2.33 | 14.83 | 8.17 | 0.50 | 0.50 |
| HSPG2 | Basement membrane-specific heparan sulfate proteoglycan core protein | 5.33 | 1.88 | 7.00 | 2.52 | 7.83 | 3.98 |
| F2 | Prothrombin | 8.33 | 1.36 | 9.17 | 1.96 | 2.67 | 1.59 |
| RNASE4 | Ribonuclease 4 | 9.00 | 0.29 | 7.33 | 1.01 | 1.67 | 0.88 |
| LRP1 | Prolow-density lipoprotein receptor-related protein 1 | 7.33 | 0.60 | 9.33 | 1.48 | 1.00 | 0.76 |
| AMBP | Protein AMBP | 6.67 | 0.44 | 6.50 | 0.76 | 4.00 | 1.53 |
| H1-0 | Histone H1.0 | 5.00 | 3.75 | 11.67 | 6.01 | 0.00 | 0.00 |
| APOH | Beta-2-glycoprotein 1 | 7.50 | 1.26 | 6.33 | 1.86 | 2.00 | 1.04 |
| CST3 | Cystatin-C | 6.67 | 1.96 | 7.33 | 2.09 | 1.50 | 0.76 |
| FGB | Fibrinogen beta chain | 6.67 | 0.60 | 7.00 | 1.44 | 0.67 | 0.67 |
| CLU | Clusterin | 7.50 | 0.76 | 4.83 | 0.73 | 1.83 | 0.33 |
| H2BC17 | Histone H2B type 1-O | 5.83 | 0.60 | 5.83 | 1.59 | 2.17 | 1.30 |
| KRT1 | Keratin, type II cytoskeletal 1 | 6.00 | 1.73 | 3.17 | 0.67 | 3.83 | 1.30 |
| PKM | Pyruvate kinase PKM | 5.50 | 0.29 | 6.00 | 0.58 | 1.33 | 0.33 |
| CFB | Complement factor B | 7.50 | 1.26 | 4.33 | 0.17 | 0.67 | 0.33 |
| VCP | Transitional endoplasmic reticulum ATPase | 6.17 | 2.46 | 6.17 | 3.32 | 0.00 | 0.00 |
| H2AC4 | Histone H2A type 1-B/E | 5.50 | 0.50 | 6.00 | 2.02 | 0.67 | 0.44 |
| FLNA | Filamin-A | 6.33 | 0.33 | 5.33 | 0.73 | 0.17 | 0.17 |
| PLA2G2A | Phospholipase A2, membrane associated | 5.67 | 3.18 | 5.83 | 3.32 | 0.00 | 0.00 |
| GAPDH | Glyceraldehyde-3-phosphate dehydrogenase | 5.17 | 0.93 | 6.00 | 0.76 | 0.33 | 0.33 |
| CLEC11A | C-type lectin domain family 11 member A | 4.00 | 0.76 | 4.17 | 0.44 | 3.33 | 1.67 |
| APOA2 | Apolipoprotein A-II | 4.33 | 0.88 | 5.50 | 0.29 | 1.33 | 0.73 |
| SERPINF1 | Pigment epithelium-derived factor | 3.67 | 0.33 | 5.83 | 1.64 | 1.50 | 0.76 |
| DKK1 | Dickkopf-related protein 1 | 6.83 | 1.09 | 4.17 | 0.33 | 0.00 | 0.00 |
| LAMA5 | Laminin subunit alpha-5 | 5.67 | 1.01 | 4.50 | 0.76 | 0.50 | 0.50 |
| KRT9 | Keratin, type I cytoskeletal 9 | 6.17 | 1.96 | 4.17 | 0.33 | 0.17 | 0.17 |
| CFI | Complement factor I | 3.50 | 0.29 | 5.33 | 1.09 | 1.67 | 0.83 |
| GPC3 | Glypican-3 | 6.50 | 2.52 | 3.67 | 1.20 | 0.00 | 0.00 |
| H3C1 | Histone H3.1 | 5.50 | 1.44 | 4.50 | 2.08 | 0.00 | 0.00 |
| PSMA1 | Proteasome subunit alpha type-1 | 3.17 | 0.44 | 5.67 | 0.93 | 1.00 | 0.29 |
| RBP4 | Retinol-binding protein 4 | 4.17 | 0.88 | 5.17 | 0.88 | 0.50 | 0.29 |
| AGRN | Agrin | 4.50 | 0.87 | 2.83 | 0.17 | 2.33 | 1.20 |
| VTN | Vitronectin | 5.50 | 1.26 | 3.17 | 0.73 | 0.83 | 0.17 |
| TUBB4A | Tubulin beta-4A chain | 2.83 | 0.33 | 4.33 | 0.17 | 2.00 | 1.04 |
| ITIH1 | Inter-alpha-trypsin inhibitor heavy chain H1 | 2.67 | 0.44 | 4.17 | 0.17 | 2.17 | 0.88 |
| VASP | Vasodilator-stimulated phosphoprotein | 3.17 | 0.60 | 3.33 | 0.33 | 1.83 | 1.01 |
| PGAM1 | Phosphoglycerate mutase 1 | 3.67 | 0.73 | 3.00 | 0.76 | 1.67 | 0.88 |
| COL1A1 | Collagen alpha-1(I) chain | 2.50 | 0.29 | 3.00 | 0.29 | 2.67 | 0.60 |
| FGG | Fibrinogen gamma chain | 5.67 | 1.59 | 2.33 | 1.59 | 0.00 | 0.00 |
| LUM | Lumican | 3.83 | 0.73 | 2.17 | 0.44 | 2.00 | 1.15 |
| YWHAZ | 14-3-3 protein zeta/delta | 2.67 | 0.17 | 3.67 | 0.60 | 1.67 | 0.88 |
| APOC3 | Apolipoprotein C-III | 3.67 | 0.17 | 2.67 | 0.73 | 1.67 | 1.01 |
| MDK | Midkine | 2.33 | 1.45 | 5.50 | 3.50 | 0.00 | 0.00 |
| YWHAQ | 14-3-3 protein theta | 3.17 | 0.17 | 3.50 | 0.50 | 0.33 | 0.33 |
| PCSK9 | Proprotein convertase subtilisin/kexin type 9 | 5.33 | 1.17 | 1.17 | 0.33 | 0.17 | 0.17 |
| PPIA | Peptidyl-prolyl cis-trans isomerase A | 1.83 | 0.33 | 4.00 | 0.76 | 0.67 | 0.67 |
| MT2A | Metallothionein-2 | 2.33 | 0.33 | 2.50 | 0.58 | 1.67 | 0.83 |
| IGF2 | Insulin-like growth factor II | 2.33 | 0.17 | 2.50 | 0.50 | 1.33 | 0.73 |
| TFPI | Tissue factor pathway inhibitor | 3.33 | 0.17 | 1.83 | 0.60 | 1.00 | 0.00 |
| SERPINF2 | Alpha-2-antiplasmin | 2.33 | 0.60 | 2.67 | 0.60 | 1.00 | 0.50 |
| EFEMP1 | EGF-containing fibulin-like extracellular matrix protein 1 | 2.33 | 0.17 | 2.67 | 0.33 | 1.00 | 0.58 |
| LGALS3BP | Galectin-3-binding protein | 1.67 | 0.33 | 3.00 | 1.32 | 1.00 | 0.50 |
| ATP1A1 | Sodium/potassium-transporting ATPase subunit alpha-1 | 2.00 | 1.00 | 3.67 | 0.73 | 0.00 | 0.00 |
| C2 | Complement C2 | 3.67 | 0.33 | 2.00 | 0.29 | 0.00 | 0.00 |
| FBXO39 | F-box only protein 39 | 2.50 | 0.29 | 2.00 | 0.29 | 1.17 | 0.60 |
| PSMA5 | Proteasome subunit alpha type-5 | 2.67 | 1.01 | 2.83 | 1.20 | 0.17 | 0.17 |
| TGFBI | Transforming growth factor-beta-induced protein ig-h3 | 2.67 | 0.17 | 1.67 | 0.44 | 1.17 | 0.60 |
| POSTN | Periostin | 2.33 | 0.33 | 2.67 | 0.33 | 0.50 | 0.50 |
| LIMS1 | LIM and senescent cell antigen-like-containing domain protein 1 | 1.83 | 0.17 | 2.67 | 1.30 | 1.00 | 0.76 |
| FASN | Fatty acid synthase | 2.00 | 1.04 | 3.33 | 0.44 | 0.00 | 0.00 |
| KRT10 | Keratin, type I cytoskeletal 10 | 3.33 | 1.42 | 1.00 | 0.76 | 0.83 | 0.44 |
| COL1A2 | Collagen alpha-2(I) chain | 2.00 | 0.50 | 2.17 | 0.17 | 1.00 | 0.58 |
| RAN | GTP-binding nuclear protein Ran | 2.50 | 0.58 | 1.83 | 0.17 | 0.67 | 0.44 |
| DNAJB9 | DnaJ homolog subfamily B member 9 | 2.83 | 0.17 | 2.17 | 0.67 | 0.00 | 0.00 |
| SERPINA1 | Alpha-1-antitrypsin | 1.67 | 0.44 | 2.17 | 0.60 | 1.00 | 0.50 |
| DMBT1 | Deleted in malignant brain tumors 1 protein | 1.50 | 0.50 | 1.67 | 0.44 | 1.67 | 0.83 |
| H4C1 | Histone H4 | 2.17 | 1.17 | 2.50 | 1.26 | 0.00 | 0.00 |
| SLC3A2 | 4F2 cell-surface antigen heavy chain | 1.50 | 0.29 | 3.17 | 0.33 | 0.00 | 0.00 |
| TUBB1 | Tubulin beta-1 chain | 1.83 | 0.33 | 1.33 | 0.17 | 1.33 | 0.17 |
| INHBE | Inhibin beta E chain | 1.83 | 0.44 | 2.33 | 0.17 | 0.17 | 0.17 |
| COL2A1 | Collagen alpha-1(II) chain | 1.33 | 0.17 | 1.00 | 0.00 | 2.00 | 0.50 |
| SEPTIN7 | Septin-7 | 1.50 | 0.00 | 2.17 | 0.17 | 0.67 | 0.44 |
| RPS8 | 40S ribosomal protein S8 | 0.50 | 0.50 | 3.67 | 0.33 | 0.00 | 0.00 |
| SCAMP3 | Secretory carrier-associated membrane protein 3 | 1.50 | 0.29 | 2.33 | 0.17 | 0.33 | 0.33 |
| LAMB1 | Laminin subunit beta-1 | 1.67 | 0.44 | 1.50 | 0.29 | 0.83 | 0.44 |
| GDI2 | Rab GDP dissociation inhibitor beta | 2.33 | 0.44 | 1.67 | 0.33 | 0.00 | 0.00 |
| KNG1 | Kininogen-1 | 1.33 | 0.33 | 2.00 | 0.29 | 0.50 | 0.29 |
| CLEC3B | Tetranectin | 1.50 | 0.58 | 2.33 | 0.17 | 0.00 | 0.00 |
| KRT5 | Keratin, type II cytoskeletal 5 | 2.50 | 1.26 | 0.67 | 0.17 | 0.50 | 0.50 |
| C7 | Complement component C7 | 1.50 | 0.29 | 2.17 | 0.93 | 0.00 | 0.00 |
| ANG | Angiogenin | 3.33 | 0.44 | 0.33 | 0.33 | 0.00 | 0.00 |
| HP | Haptoglobin | 1.33 | 0.60 | 2.33 | 1.01 | 0.00 | 0.00 |
| PCSK6 | Proprotein convertase subtilisin/kexin type 6 | 1.33 | 0.60 | 1.67 | 0.17 | 0.50 | 0.50 |
| F5 | Coagulation factor V | 1.67 | 0.67 | 1.67 | 0.33 | 0.17 | 0.17 |
| SPP1 | Osteopontin | 1.17 | 0.17 | 1.50 | 0.29 | 0.83 | 0.44 |
| TIMP1 | Metalloproteinase inhibitor 1 | 1.17 | 0.93 | 1.83 | 0.17 | 0.50 | 0.50 |
| SDCBP | Syntenin-1 | 1.67 | 0.17 | 1.50 | 0.29 | 0.33 | 0.33 |
| TUBB | Tubulin beta chain | 1.17 | 0.44 | 1.67 | 0.67 | 0.50 | 0.29 |
| KRT6A | Keratin, type II cytoskeletal 6A | 1.83 | 1.17 | 1.00 | 0.00 | 0.50 | 0.29 |
| VCL | Vinculin | 1.00 | 0.00 | 1.17 | 0.17 | 1.17 | 0.60 |
| ILK | Integrin-linked protein kinase | 1.50 | 0.50 | 1.50 | 0.58 | 0.17 | 0.17 |
| ENO1 | Alpha-enolase | 2.00 | 0.29 | 1.17 | 0.67 | 0.00 | 0.00 |
| RARRES2 | Retinoic acid receptor responder protein 2 | 1.17 | 0.44 | 2.00 | 1.26 | 0.00 | 0.00 |
| HSPA8 | Heat shock cognate 71 kDa protein | 1.33 | 0.33 | 1.33 | 0.33 | 0.50 | 0.29 |
| SPINK1 | Serine protease inhibitor Kazal-type 1 | 1.00 | 0.58 | 2.17 | 0.44 | 0.00 | 0.00 |
| THBS4 | Thrombospondin-4 | 1.17 | 0.67 | 1.67 | 0.44 | 0.17 | 0.17 |
| SUB1 | Activated RNA polymerase II transcriptional coactivator p15 | 0.83 | 0.83 | 2.17 | 1.09 | 0.00 | 0.00 |
| XRCC6 | X-ray repair cross-complementing protein 6 | 1.00 | 1.00 | 2.00 | 1.04 | 0.00 | 0.00 |
| DNAH8 | Dynein axonemal heavy chain 8 | 1.17 | 0.73 | 0.50 | 0.00 | 1.33 | 0.67 |
| DPYSL2 | Dihydropyrimidinase-related protein 2 | 1.67 | 0.44 | 1.17 | 0.73 | 0.00 | 0.00 |
| B4GAT1 | Beta-1,4-glucuronyltransferase 1 | 1.00 | 0.00 | 1.17 | 0.44 | 0.67 | 0.33 |
| ALDH16A1 | Aldehyde dehydrogenase family 16 member A1 | 1.00 | 0.00 | 1.50 | 0.50 | 0.33 | 0.33 |
| PZP | Pregnancy zone protein | 1.33 | 0.33 | 1.17 | 0.44 | 0.17 | 0.17 |
| LAMC1 | Laminin subunit gamma-1 | 0.67 | 0.17 | 2.00 | 1.00 | 0.00 | 0.00 |
| CD44 | CD44 antigen | 1.50 | 0.00 | 0.67 | 0.33 | 0.50 | 0.29 |
| HINT1 | Adenosine 5'-monophosphoramidase HINT1 | 1.50 | 0.29 | 0.83 | 0.17 | 0.33 | 0.17 |
| NPM1 | Nucleophosmin | 0.33 | 0.17 | 2.33 | 0.33 | 0.00 | 0.00 |
| MIF | Macrophage migration inhibitory factor | 1.50 | 0.87 | 1.17 | 0.44 | 0.00 | 0.00 |
| EEF1A1P5 | Putative elongation factor 1-alpha-like 3 | 0.67 | 0.33 | 1.50 | 0.00 | 0.33 | 0.33 |
| MVP | Major vault protein | 0.00 | 0.00 | 2.50 | 0.29 | 0.00 | 0.00 |
| B2M | Beta-2-microglobulin | 1.00 | 0.00 | 1.50 | 0.29 | 0.00 | 0.00 |
| C8B | Complement component C8 beta chain | 1.00 | 0.00 | 1.17 | 0.17 | 0.33 | 0.17 |
| SEPTIN2 | Septin-2 | 0.50 | 0.29 | 2.00 | 0.76 | 0.00 | 0.00 |
| H2AC20 | Histone H2A type 2-C | 1.00 | 0.29 | 1.33 | 0.60 | 0.00 | 0.00 |
| ELOB | Elongin-B | 1.00 | 0.76 | 0.67 | 0.17 | 0.67 | 0.44 |
| NOTUM | Palmitoleoyl-protein carboxylesterase NOTUM | 0.33 | 0.33 | 1.83 | 0.93 | 0.00 | 0.00 |
| RPL5 | 60S ribosomal protein L5 | 0.83 | 0.44 | 1.33 | 0.17 | 0.00 | 0.00 |
| H1-5 | Histone H1.5 | 0.17 | 0.17 | 1.83 | 1.83 | 0.00 | 0.00 |
| CLTC | Clathrin heavy chain 1 | 0.00 | 0.00 | 2.00 | 0.50 | 0.00 | 0.00 |
| CCT3 | T-complex protein 1 subunit gamma | 0.83 | 0.44 | 1.17 | 0.17 | 0.00 | 0.00 |
| HAMP | Hepcidin | 0.00 | 0.00 | 0.17 | 0.17 | 1.83 | 0.93 |
| MIS18A | Protein Mis18-alpha | 1.17 | 0.33 | 0.67 | 0.44 | 0.17 | 0.17 |
| CPN2 | Carboxypeptidase N subunit 2 | 0.33 | 0.33 | 1.67 | 1.17 | 0.00 | 0.00 |
| RAP1BL | Ras-related protein Rap-1b-like protein | 0.67 | 0.44 | 0.83 | 0.17 | 0.33 | 0.33 |
| OGN | Mimecan | 0.33 | 0.17 | 0.67 | 0.17 | 0.83 | 0.60 |
| FRAS1 | Extracellular matrix organizing protein FRAS1 | 1.17 | 0.67 | 0.67 | 0.44 | 0.00 | 0.00 |
| UGDH | UDP-glucose 6-dehydrogenase | 0.33 | 0.33 | 1.17 | 0.17 | 0.17 | 0.17 |
| NID1 | Nidogen-1 | 0.83 | 0.33 | 0.67 | 0.67 | 0.00 | 0.00 |
| SERPINC1 | Antithrombin-III | 0.17 | 0.17 | 1.00 | 0.29 | 0.33 | 0.17 |
| RPL7 | 60S ribosomal protein L7 | 0.17 | 0.17 | 1.33 | 0.73 | 0.00 | 0.00 |
| PLG | Plasminogen | 0.50 | 0.29 | 1.00 | 0.58 | 0.00 | 0.00 |
| EIF4A3 | Eukaryotic initiation factor 4A-III | 0.00 | 0.00 | 1.33 | 0.33 | 0.17 | 0.17 |
| RNASET2 | Ribonuclease T2 | 1.33 | 0.17 | 0.00 | 0.00 | 0.17 | 0.17 |
| CCT6A | T-complex protein 1 subunit zeta | 0.00 | 0.00 | 1.50 | 0.76 | 0.00 | 0.00 |
| CX3CL1 | Fractalkine | 0.83 | 0.33 | 0.67 | 0.44 | 0.00 | 0.00 |
| LTF | Lactotransferrin | 1.17 | 0.17 | 0.33 | 0.33 | 0.00 | 0.00 |
| IGLC2 | Immunoglobulin lambda constant 2 | 0.50 | 0.50 | 0.00 | 0.00 | 0.83 | 0.83 |
| ITIH3 | Inter-alpha-trypsin inhibitor heavy chain H3 | 0.33 | 0.17 | 0.33 | 0.33 | 0.67 | 0.67 |
| EEF2 | Elongation factor 2 | 0.17 | 0.17 | 1.00 | 0.29 | 0.17 | 0.17 |
| FSTL3 | Follistatin-related protein 3 | 0.00 | 0.00 | 1.33 | 0.83 | 0.00 | 0.00 |
| VASN | Vasorin | 0.17 | 0.17 | 1.17 | 0.93 | 0.00 | 0.00 |
| PSAP | Prosaposin | 0.33 | 0.33 | 1.00 | 1.00 | 0.00 | 0.00 |
| PVR | Poliovirus receptor | 0.83 | 0.33 | 0.50 | 0.29 | 0.00 | 0.00 |
| APOA5 | Apolipoprotein A-V | 1.17 | 0.60 | 0.00 | 0.00 | 0.00 | 0.00 |
| SRSF6 | Serine/arginine-rich splicing factor 6 | 0.00 | 0.00 | 1.17 | 0.60 | 0.00 | 0.00 |
| HSPD1 | 60 kDa heat shock protein, mitochondrial | 0.33 | 0.33 | 0.83 | 0.33 | 0.00 | 0.00 |
| TUBB8B | Tubulin beta 8B | 0.50 | 0.29 | 0.67 | 0.33 | 0.00 | 0.00 |
| FMOD | Fibromodulin | 0.50 | 0.29 | 0.67 | 0.44 | 0.00 | 0.00 |
| PTTG1IP | Pituitary tumor-transforming gene 1 protein-interacting protein | 1.17 | 0.93 | 0.00 | 0.00 | 0.00 | 0.00 |
| KRT2 | Keratin, type II cytoskeletal 2 epidermal | 0.67 | 0.33 | 0.17 | 0.17 | 0.17 | 0.17 |
| H2AZ1 | Histone H2A.Z | 0.00 | 0.00 | 1.00 | 1.00 | 0.00 | 0.00 |
| IGF1 | Insulin-like growth factor I | 0.33 | 0.17 | 0.33 | 0.33 | 0.33 | 0.33 |
| RPS2 | 40S ribosomal protein S2 | 0.00 | 0.00 | 1.00 | 0.58 | 0.00 | 0.00 |
| PSMB6 | Proteasome subunit beta type-6 | 0.17 | 0.17 | 0.83 | 0.33 | 0.00 | 0.00 |
| TIE1 | Tyrosine-protein kinase receptor Tie-1 | 0.33 | 0.17 | 0.33 | 0.17 | 0.33 | 0.33 |
| RPL13 | 60S ribosomal protein L13 | 0.00 | 0.00 | 1.00 | 0.29 | 0.00 | 0.00 |
| SF3B5 | Splicing factor 3B subunit 5 | 0.00 | 0.00 | 1.00 | 0.58 | 0.00 | 0.00 |
| C9 | Complement component C9 | 0.17 | 0.17 | 0.83 | 0.17 | 0.00 | 0.00 |
| TNFRSF12A | Tumor necrosis factor receptor superfamily member 12A | 0.17 | 0.17 | 0.83 | 0.33 | 0.00 | 0.00 |
| RPL28 | 60S ribosomal protein L28 | 0.17 | 0.17 | 0.83 | 0.17 | 0.00 | 0.00 |
| HMGA1 | High mobility group protein HMG-I/HMG-Y | 0.17 | 0.17 | 0.83 | 0.60 | 0.00 | 0.00 |
| PLXNB2 | Plexin-B2 | 0.33 | 0.17 | 0.67 | 0.33 | 0.00 | 0.00 |
| APOM | Apolipoprotein M | 1.00 | 0.50 | 0.00 | 0.00 | 0.00 | 0.00 |
| TUBA1B | Tubulin alpha-1B chain | 0.17 | 0.17 | 0.50 | 0.29 | 0.17 | 0.17 |
| WDR1 | WD repeat-containing protein 1 | 0.00 | 0.00 | 0.83 | 0.44 | 0.00 | 0.00 |
| RPL3 | 60S ribosomal protein L3 | 0.00 | 0.00 | 0.83 | 0.17 | 0.00 | 0.00 |
| CDH2 | Cadherin-2 | 0.83 | 0.44 | 0.00 | 0.00 | 0.00 | 0.00 |
| RPS11 | 40S ribosomal protein S11 | 0.00 | 0.00 | 0.67 | 0.33 | 0.00 | 0.00 |
| C4BPA | C4b-binding protein alpha chain | 0.00 | 0.00 | 0.67 | 0.67 | 0.00 | 0.00 |
| PSMA6 | Proteasome subunit alpha type-6 | 0.00 | 0.00 | 0.67 | 0.33 | 0.00 | 0.00 |
| IGFBP5 | Insulin-like growth factor-binding protein 5 | 0.33 | 0.17 | 0.33 | 0.17 | 0.00 | 0.00 |
| COL18A1 | Collagen alpha-1(XVIII) chain | 0.33 | 0.17 | 0.33 | 0.17 | 0.00 | 0.00 |
| ADAMTS13 | A disintegrin and metalloproteinase with thrombospondin motifs 13 | 0.67 | 0.44 | 0.00 | 0.00 | 0.00 | 0.00 |
| TUBB3 | Tubulin beta-3 chain | 0.00 | 0.00 | 0.50 | 0.29 | 0.00 | 0.00 |
| MAN1A1 | Mannosyl-oligosaccharide 1,2-alpha-mannosidase IA | 0.33 | 0.33 | 0.17 | 0.17 | 0.00 | 0.00 |
| GDF15 | Growth/differentiation factor 15 | 0.00 | 0.00 | 0.50 | 0.50 | 0.00 | 0.00 |
| CCN2 | CCN family member 2 | 0.50 | 0.50 | 0.00 | 0.00 | 0.00 | 0.00 |
| IGKC | Immunoglobulin kappa constant | 0.00 | 0.00 | 0.00 | 0.00 | 0.50 | 0.50 |
| IGHG2 | Immunoglobulin heavy constant gamma 2 | 0.00 | 0.00 | 0.00 | 0.00 | 0.50 | 0.50 |
| IGHM | Immunoglobulin heavy constant mu | 0.00 | 0.00 | 0.00 | 0.00 | 0.50 | 0.50 |
| VCAN | Versican core protein | 0.00 | 0.00 | 0.00 | 0.00 | 0.50 | 0.29 |
| ACAN | Aggrecan core protein | 0.17 | 0.17 | 0.00 | 0.00 | 0.33 | 0.33 |
| MYH9 | Myosin-9 | 0.00 | 0.00 | 0.33 | 0.17 | 0.17 | 0.17 |
| FSCN1 | Fascin | 0.00 | 0.00 | 0.50 | 0.29 | 0.00 | 0.00 |
| HP1BP3 | Heterochromatin protein 1-binding protein 3 | 0.00 | 0.00 | 0.50 | 0.50 | 0.00 | 0.00 |
| TWSG1 | Twisted gastrulation protein homolog 1 | 0.00 | 0.00 | 0.50 | 0.29 | 0.00 | 0.00 |
| COL16A1 | Collagen alpha-1(XVI) chain | 0.17 | 0.17 | 0.33 | 0.33 | 0.00 | 0.00 |
| RPL15 | 60S ribosomal protein L15 | 0.17 | 0.17 | 0.33 | 0.33 | 0.00 | 0.00 |
| CCT5 | T-complex protein 1 subunit epsilon | 0.00 | 0.00 | 0.33 | 0.33 | 0.00 | 0.00 |
| TSSK4 | Testis-specific serine/threonine-protein kinase 4 | 0.00 | 0.00 | 0.00 | 0.00 | 0.33 | 0.17 |
| PGK1 | Phosphoglycerate kinase 1 | 0.00 | 0.00 | 0.33 | 0.17 | 0.00 | 0.00 |
| CORO1B | Coronin-1B | 0.00 | 0.00 | 0.33 | 0.33 | 0.00 | 0.00 |
| CLTA | Clathrin light chain A | 0.00 | 0.00 | 0.33 | 0.33 | 0.00 | 0.00 |
| COPA | Coatomer subunit alpha | 0.00 | 0.00 | 0.33 | 0.33 | 0.00 | 0.00 |
| CAT | Catalase | 0.00 | 0.00 | 0.33 | 0.17 | 0.00 | 0.00 |
| CDH6 | Cadherin-6 | 0.00 | 0.00 | 0.33 | 0.17 | 0.00 | 0.00 |
| CD59 | CD59 glycoprotein | 0.00 | 0.00 | 0.33 | 0.33 | 0.00 | 0.00 |
| RPS6 | 40S ribosomal protein S6 | 0.00 | 0.00 | 0.33 | 0.33 | 0.00 | 0.00 |
| HPX | Hemopexin | 0.17 | 0.17 | 0.17 | 0.17 | 0.00 | 0.00 |
| CTSB | Cathepsin B | 0.17 | 0.17 | 0.17 | 0.17 | 0.00 | 0.00 |
| TUBB4B | Tubulin beta-4B chain | 0.00 | 0.00 | 0.17 | 0.17 | 0.00 | 0.00 |
| VIM | Vimentin | 0.00 | 0.00 | 0.00 | 0.00 | 0.17 | 0.17 |
| PCSK1N | ProSAAS | 0.00 | 0.00 | 0.17 | 0.17 | 0.00 | 0.00 |
| IGF2R | Cation-independent mannose-6-phosphate receptor | 0.00 | 0.00 | 0.17 | 0.17 | 0.00 | 0.00 |
| AHCY | Adenosylhomocysteinase | 0.00 | 0.00 | 0.17 | 0.17 | 0.00 | 0.00 |
| ALDH1A1 | Aldehyde dehydrogenase 1A1 | 0.00 | 0.00 | 0.17 | 0.17 | 0.00 | 0.00 |
| PCNA | Proliferating cell nuclear antigen | 0.00 | 0.00 | 0.17 | 0.17 | 0.00 | 0.00 |
| P4HA1 | Prolyl 4-hydroxylase subunit alpha-1 | 0.00 | 0.00 | 0.17 | 0.17 | 0.00 | 0.00 |
| LAMA2 | Laminin subunit alpha-2 | 0.00 | 0.00 | 0.17 | 0.17 | 0.00 | 0.00 |
| HNRNPM | Heterogeneous nuclear ribonucleoprotein M | 0.00 | 0.00 | 0.17 | 0.17 | 0.00 | 0.00 |
| HNRNPF | Heterogeneous nuclear ribonucleoprotein F | 0.00 | 0.00 | 0.17 | 0.17 | 0.00 | 0.00 |
| RUVBL2 | RuvB-like 2 | 0.00 | 0.00 | 0.17 | 0.17 | 0.00 | 0.00 |
| CKAP5 | Cytoskeleton-associated protein 5 | 0.17 | 0.17 | 0.00 | 0.00 | 0.00 | 0.00 |
| FETUB | Fetuin-B | 0.17 | 0.17 | 0.00 | 0.00 | 0.00 | 0.00 |
| LOX | Protein-lysine 6-oxidase | 0.17 | 0.17 | 0.00 | 0.00 | 0.00 | 0.00 |
| APOB | Apolipoprotein B-100 | 0.17 | 0.17 | 0.00 | 0.00 | 0.00 | 0.00 |
| NDRG1 | Protein NDRG1 | 0.17 | 0.17 | 0.00 | 0.00 | 0.00 | 0.00 |
| TMEM123 | Porimin | 0.17 | 0.17 | 0.00 | 0.00 | 0.00 | 0.00 |
